# Barriers to contraception access and use among youth: A scoping review in high‐income countries

**DOI:** 10.1002/ijgo.70637

**Published:** 2025-11-14

**Authors:** Bronte K. Johnston, Patricia A. Janssen, Mika Ohtsuka, Zeba Khan, Ciara Madden, Chelsey Perry, Piper Scott‐Fiddler, Sarah Munro, Laura Schummers, Kimberlyn M. McGrail

**Affiliations:** ^1^ School of Population and Public Health The University of British Columbia Vancouver British Columbia Canada; ^2^ Department of Obstetrics and Gynecology The University of British Columbia Vancouver British Columbia Canada; ^3^ Department of Health Systems and Population Health University of Washington Washington USA; ^4^ Collaboration for Outcomes Research and Evaluation The University of British Columbia Vancouver British Columbia Canada

**Keywords:** adolescent, contraception, contraceptive agents, health services accessibility, high‐income countries, youth

## Abstract

**Background:**

The United Nations (UN) has a target for universal contraception access by 2030. Youth (aged 15–29) still have limited contraception access and lower usage. A unified understanding of the barriers youth face in high‐income countries (HIC) remains unclear.

**Objectives:**

Synthesized evidence on youth contraception barriers across HIC to identify continued healthcare inaccessibility and knowledge gaps.

**Search Strategy:**

A search strategy, including terms like “youth” and “barriers,” was applied to three databases, identifying articles published between January 2013–September 2024.

**Selection Criteria:**

Primary peer‐reviewed quantitative, qualitative, and mixed‐methods studies were included if they focused on youth and contraception barriers.

**Data Collection and Analysis:**

Following the Joanna Briggs Institute, articles were screened for inclusion, and data was extracted. Analyses included descriptive statistics and summarizing findings for quantitative and qualitative results. All articles were subjected to inductive and deductive content analysis to map barriers. Article quality was appraised by the Mixed Methods Appraisal Tool.

**Main Results:**

A total of 41 articles were included, of which 88% were from the USA. Youth struggled to receive quality contraception care from multiple access points from health systems and youth perspectives. Barriers included youth minimal knowledge, poor approachability and care appropriateness, physical barriers, costs, stigma, confidentiality concerns, and service gatekeeping. Youth experiences varied by social identities with those from lower economic, rural, and of younger age facing more obstacles.

**Conclusions:**

Contraception was inaccessible for many. To meet UN targets, efforts need to address described barriers to ensure accessible and equitable contraception care that respects and supports youth's choices.

## 1 INTRODUCTION

### 1.1 Background

The United Nations (UN) has a target for global universal contraception access by 2030 as part of a commitment to sexual and reproductive healthcare for all.^1^ Access to contraception is a human right. For youth, the ability to make reproductive health choices affects their ability to reach their personal, economic, and educational goals, and promotes gender equity.^2^ Contraception also supports patients' wellbeing beyond pregnancy control, such as sexually transmitted infection prevention, menstruation management, and polycystic ovarian syndrome and endometriosis symptom relief.^3^

Contraception uptake and accessibility are still poor in underserved populations including youth (aged 15–29).^4^, ^5^, ^6^ Sustainable change to improve access requires understanding specific contraception needs that include youth perspectives, experiences, and variations.^4^, ^5^, ^7^, ^8^ Barriers to contraception access and use among youth are an important aspect of this expanded understanding.

Known contraception barriers include cost, trust in method, inconsistent sexual health education, practitioner bias against contraception usage, and insufficient healthcare worker training.^9^, ^10^ In Canada, usage varies across social identities such as young age, socioeconomic status (SES), and rurality, which suggests that one's collective identity shapes contraception needs and access.^9^, ^11^ In the USA, racialized youth experience more barriers compared to White counterparts because of systemic medical racism that impedes the quality, accessibility, and cultural sensitivity of care.^5^, ^12^ A person's identity impacts their ability to access contraception and adequate care and therefore needs to be further understood and considered to improve contraception equity.

Existing syntheses on youth contraception barriers have predominantly focused on low‐ and middle‐income countries (LMIC).^13^, ^14^, ^15^ Published systematic reviews in high‐income countries (HIC) have concentrated on specific care approaches and groups of youth. These studies outlined the need for confidential youth‐focused contraception care, providing quality care for LGBTQIA youth, and partner influence.^16^, ^17^, ^18^ However, variations in contraception barriers among youth, and the influence of intersecting identities on those barriers, remain unclear. The dynamics of youth experiences and the roles of health and social systems in supporting or limiting contraception access are also not well understood.

### 1.2 Objective

The purpose of this review was to understand recent literature pertaining to youth contraception barriers within HIC. These findings will lay the groundwork for future research and healthcare initiatives focused on improving youth contraception equity.

## 2 METHODS

A scoping review was chosen to broadly synthesize contraception barriers to inform areas for future research and policy. The protocol is published in INPLASY (INPLASY2023100071).^19^ The present study followed the Joanna Briggs Institute (JBI) methodology for scoping reviews.^20^, ^21^ This review was conducted in accordance with the Preferred Reporting Items for Systematic reviews and Meta‐Analysis scoping review (PRISMA‐ScR) guidelines (Supplemental information 1).^22^

### 2.1 Study terms

A contraception barrier was defined as the inaccessibility of desired contraception methods and healthcare that support a person's reproductive choices. HIC includes countries with developed economies as per the 2022 World Economic Situation and Prospects.^23^ Youth were those aged 15–29 as per Statistics Canada.^6^ This definition was chosen over the WHO age range of 15–24 to recognize potential regional differences of youth classification.^24^

### 2.2 Theoretical approaches and frameworks

Reproductive justice, intersectionality, and Levesque's patient‐centered access to care framework provided complementary lenses to comprehensively understand the complexities of contraception barriers.^25^, ^26^, ^27^, ^28^

**FIGURE 1:**
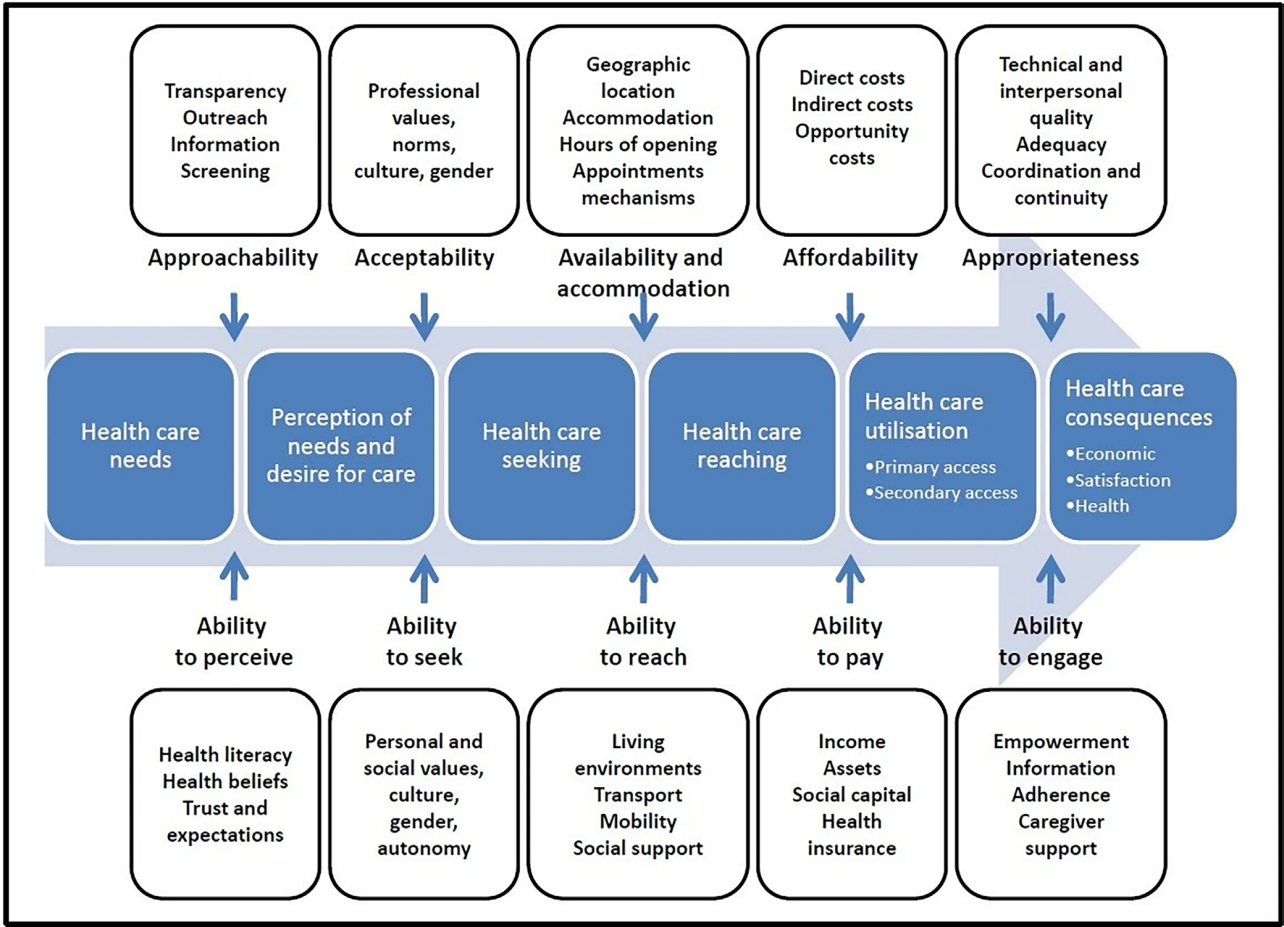
A copy of the Levesque framework to patient centered healthcare access.^27^

Reproductive justice focuses on accessibility of reproductive healthcare rather than legal rights, as rights do not guarantee access to ensure bodily autonomy and beneficence.^25^, ^28^ Intersectionality outlines how a person's experience, such as their healthcare interactions, is affected by their layered social identities and circumstances.^26^ The Levesque access to care framework emphasizes the supply and quality of healthcare as well as the demand‐side of the patient experiences which collectively impact healthcare experiences (Figure 1).^27^

### 2.3 Eligibility criteria

Studies required: (1) Youth as the primary population of focus. (2) Primary research questions/objectives focused on contraception barriers and youth. (3) Full‐text primary articles in English or French, and (4) published between January 2013 and September 2024. Quantitative, qualitative, and mixed‐methods designs were all eligible for inclusion. Unpublished, non‐peer reviewed, literature syntheses, and other non‐primary research articles, such as commentaries, were excluded.

### 2.4 Information sources

A search strategy was developed with support from a UBC librarian. Databases MEDLINE (Ovid), EMBASE (Ovid), and CINAHL were searched September 18, 2023, and September 21, 2024 (Supplemental information 2).

### 2.5 Study selection

Reviewers (BKJ, MO, CP, and ZK) screened titles and abstracts. Two reviewers read each article separately, the decision to retain or remove research articles required agreement. Articles retained for full‐text review were read by two reviewers independently and group consensus determined inclusion. All discrepancies were resolved through reviewer team discussion.

### 2.6 Data extraction

Data extraction was completed by BKJ based on a developed and iteratively updated sheet in Covidence. We extracted study design, key findings, approaches to data collection, and analytic approach. Youth demographics were noted based on the PROGRESS‐Plus tool to conceptualize different measures of health equity.^29^ We recorded if articles applied any intersectional approaches or mentioned words like intersectionality.^26^

### 2.7 Assessment of risk of bias

Article quality was appraised through the mixed methods appraisal tool (MMAT) version 2018^30^ in Covidence. Assessors (BKJ, PSF, and CM) completed the MMAT checklist for each study. Two assessors independently reviewed each article; disagreements were discussed among assessors until consensus. MMAT scores were tabulated, with mixed‐methods studies assigned an overall quality rating based on the lowest scoring component. A low score was a maximum of 40% quality criteria met.^31^ All studies were included regardless of score to fully understand the literature scope. Throughout the results, low and high scores were noted to contextualize the quality of evidence and if results were similar regardless of quality to illustrate the strength of findings.

### 2.8 Data synthesis and presentation

Study characteristics were summarized through descriptive statistics. Quantitative findings were analyzed descriptively, and qualitative studies were grouped by primary research topic to evaluate common themes. Mixed‐methods studies were summarized, analyzed descriptively, and identified if the findings converged or diverged between methods.

Qualitative content analysis was completed for all articles to identify youth contraception barrier themes.^21^, ^32^ Inductive and deductive approaches were done as described by Elo and Kyngäs^33^ and highlighted by JBI.^32^ Open coding involved inductively reviewing the extracted barriers in Microsoft Excel. Open codes were then categorized; code organization and categorization were done in Microsoft Excel and PowerPoint.^32^, ^33^ During qualitative code organization, proposed categories aligned with Levesque's patient‐centered access to care framework.^27^ During analysis, connections between the Levesque framework and described contraception barriers became apparent. Following this, proposed categories were deductively organized by adopting the Levesque framework to represent the various barriers that youth experience when trying to access contraception.^27^

## 3 RESULTS

### 3.1 Study selection

**FIGURE 2:**
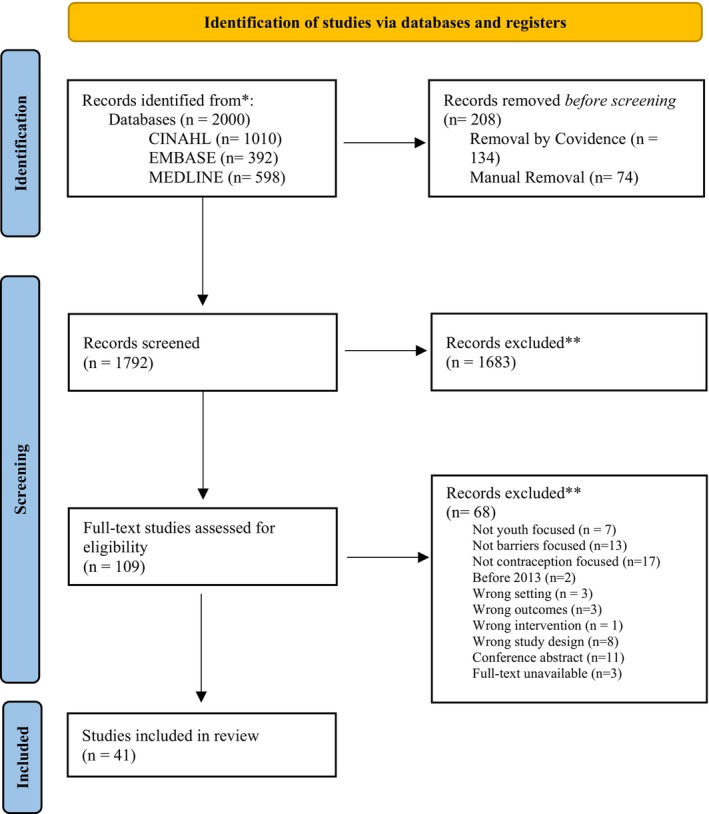
The Preferred Reporting Items for Systematic Reviews and Meta‐Analyses (PRISMA) flow diagram to determine article inclusion in a scoping review outlining youth contraception barriers in high‐income countries.^34^ *Source*: Page et al. *BMJ* 2021;372:n71. https://doi.org/10.1136/bmj.n71. This work is licensed under CC BY 4.0. To view a copy of this license, visit https://creativecommons.org/licenses/by/4.0/.

Search strategies identified 2000 articles with 208 duplications. Of the 1792 records screened, 1683 articles were excluded during title and abstract review. A total of 109 full‐text articles were reviewed, of which 41 unique primary articles were included (Figure 2,^34^ Table S1).

### 3.2 Study characteristics

There were 20 qualitative (51%), 15 quantitative (37%), and six mixed‐methods (15%) studies. Almost all studies (90%) were cross‐sectional; the most frequent data collection approaches were interviews (39%) and quantitative surveys (21%). There were four (9.8%) mystery‐caller studies, where members of the research team posed as youth inquiring about contraception. A total of 36 (88%) articles were from the USA. Most studies included only youth participants (66%), some studies included healthcare workers (20%), administrators (7.3%), parents/guardians (4.9%), and other community members (2.4%) (Table 1).

**TABLE 1:** Study characteristics of included studies in this scoping review exploring youth contraception barriers in high income countries (*n* = 41).

	*n* (%)
All	41 (100)
Overall study type	
Quantitative	15 (37)
Qualitative	20 (49)
Mixed or multimethods	6 (16)
Study design	
Cross‐sectional studies	38 (93)
Quantitative	14 (37)
Qualitative	18 (47)
Mixed or multimethods	6 (16)
Longitudinal qualitative research	2 (4.9)
Non‐randomized experimental study	1 (2.4)
Study data source	
Electronic medical records review and quantitative survey	1 (2.4)
Focus groups	3 (7.3)
Interviews	16 (39)
Interviews and focus groups	1 (2.4)
Mixed methods survey and geomapping	1 (2.4)
Mixed methods survey	3 (7.3)
Quantitative data from interviews	1 (2.4)
Quantitative survey	8 (21)
Quantitative data from written survey	1 (2.4)
Mystery caller	3 (7.3)
Mystery caller and geography analyses	1 (2.4)
Quantitative survey and qualitative interview	2 (4.9)
Geographic location	
Australia and/or New Zealand	2 (4.9)
Canada	1 (2.4)
Switzerland	1 (2.4)
UK	1 (2.4)
USA	36 (88)
Publication year	
2013–2015	5 (12)
2016–2018	10 (24)
2019–2021	19 (46)
2022–2024	7 (17)
Study participants	
Youth	27 (66)
Youth in general	4 (15)
Female youth only	17 (63)
Indigenous female youth	1 (3.7)
Commercially sexually exploited female youth	1 (3.7)
Male youth only	1 (3.7)
Homeless youth	3 (11)
Healthcare workers	8 (20)
Pharmacists and pharmacy staff	3 (38)
Physicians	2 (25)
General healthcare practitioners	1 (13)
High school nurses	1 (13)
Various healthcare workers	1 (13)
Administrators	6 (15)
Health administrators	3 (50)
Practice/program administrators	2 (33)
Juvenile justice state level health administrators youth	1 (17)
Parents and guardians	1 (2.4)
Other or general youth sexual and reproductive health Community members	1 (2.4)
Youth and caregivers	1 (2.4)

*Note*: Percentages were calculated column‐wise based on the denominator of all articles (*n* = 41).

### 3.3 Risk of bias of included studies

A total of 21 (51%) articles had a 100% MMAT score for their respective study design (qualitative *n* = 17 [85%]; quantitative *n* = 4 [27%]; mixed‐methods *n* = 0 [0.0%]).^30^, ^35^, ^36^, ^37^, ^38^, ^39^, ^40^, ^41^, ^42^, ^43^, ^44^, ^45^, ^46^, ^47^, ^48^, ^49^, ^50^, ^51^, ^52^, ^53^, ^54^ Eight studies scored 40% or lower.^55^, ^56^, ^57^, ^58^, ^59^, ^60^, ^61^, ^62^ Lower scores were commonly a result of inadequate description of study methods. Areas of strength included appropriate sampling strategies, clear description of study measures utilized, and data interpretation.^35^, ^36^, ^37^, ^38^, ^39^, ^40^, ^41^, ^42^, ^43^, ^44^, ^45^, ^46^, ^47^, ^48^, ^49^, ^50^, ^51^, ^52^, ^53^, ^54^ Mixed‐methods articles were primarily quantitative‐focused with a small qualitative component. These studies did not clearly present their rationale for study design, and many did not adequately integrate quantitative and qualitative findings.^51^, ^56^, ^57^, ^59^, ^60^, ^61^ (Tables S2A).

### 3.4 Participant demographics

Youth were primarily living or had the ability to travel to urban communities (44%). Two studies were situated in rural settings, six were in mixed community sizes, and 17 did not state the study setting. All articles reported on their sex or gender; 11 articles specified sex or gender or included language like gender assigned at birth; most study participants were classified as female. Participants were asked about their sexual orientation explicitly in seven studies.^36^, ^39^, ^41^, ^50^, ^60^, ^63^, ^64^ When asked, most youth identified as heterosexual or were sexually active with males. Few participants identified as homosexual, pansexual, or bisexual.^36^, ^39^, ^41^, ^50^, ^60^, ^63^, ^64^ Three studies required participants to be previously, currently, or intended to engage in a heterosexual sexual partnership.^42^, ^55^, ^65^ Youth encompassed a diversity of racial, ethnic, and Indigenous identities. Frequently reported identities included White, Black and Hispanic/Latinx.^36^, ^39^, ^41^, ^42^, ^45^, ^46^, ^47^, ^49^, ^50^, ^51^, ^54^, ^55^, ^59^, ^60^, ^63^, ^64^, ^65^, ^66^, ^67^, ^68^, ^69^, ^70^, ^71^, ^72^ SES, health insurance, and income were reported in 22 articles, reporting more youth from low‐income backgrounds. Youth were either dependent on their parental insurance or without insurance.^35^, ^36^, ^39^, ^42^, ^46^, ^50^, ^51^, ^54^, ^55^, ^57^, ^58^, ^59^, ^60^, ^63^, ^64^, ^65^, ^66^, ^67^, ^68^, ^69^, ^72^, ^73^ Most youth reported being sexually active when asked.^41^, ^42^, ^55^, ^58^, ^59^, ^63^, ^65^, ^66^, ^67^, ^68^, ^69^, ^73^ Youth were enrolled or completed high school or postsecondary education and had work experience.^41^, ^47^, ^53^, ^54^, ^56^, ^57^, ^58^, ^63^, ^64^, ^65^, ^68^, ^69^, ^73^ Minimal information regarding youth immigration status or preferred language was available. Among the few studies that collected and reported these demographics, youth preferred the English or Spanish language, and large proportions of the study sample were immigrants (if birth country was specified, youth were from North and South America)^49^, ^53^, ^57^, ^58^ (Table S3).

Only three studies (7.3%) directly referred to intersectionality, one of these articles had a low MMAT score. Studies referred to intersectionality with respect to the need for more research including social determinants of health or explored the connections between youth identities and experiences with contraception choices.^36^, ^39^, ^59^ Intersectionality was indirectly referenced through discussion of reproductive justice and the need to recognize oppression experienced by marginalized communities.^71^

### 3.5 Synthesis of results

**FIGURE 3:**
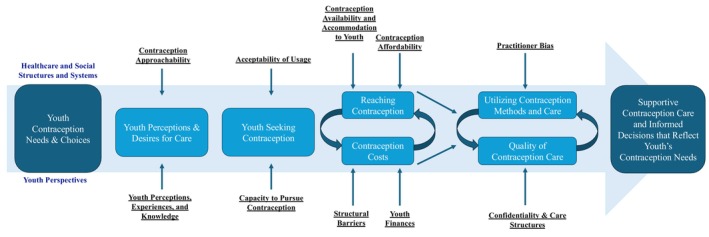
Youth contraception barriers framework, based on findings from a scoping review outlining youth contraception barriers in high‐income countries from 2013 to 2024 (*n* = 41). Adapted from the Levesque framework to patient centered healthcare access.^27^

This scoping review identified eight themes, which generally aligned with the Levesque framework (Figure 1).^27^ The themes specifically related to contraception barriers for youth included: needs and choices; perceptions and desires; seeking; reaching; costs; quality and supportive care. These are shown in an adapted framework (Figure 3).^27^ Based on their needs, youth perceived their desires for care, which then impacted their actions to seek care. Their ability to reach and afford care influenced whether youth utilized and subsequently received quality care that enabled informed contraception decisions. While this framework is linear, youth could move among access barriers. Each of these themes is expanded below.

#### 3.5.1 Youth contraception needs and choices

Findings focused on the dynamics of youth individually as well as the larger healthcare and social structures and available healthcare resources that affect how youth identified their contraception needs and choices. The journey to receiving quality care that reflected youths reproductive health needs was dependent on them first identifying those needs, which then shaped their interactions with social and healthcare systems.

##### 3.5.1.1 Preferred method

Types of contraception included both hormonal and non‐hormonal methods. Given the diversity of methods, youth had specific contraception priorities that best reflected their reproductive health goals, but access was often challenging. Five articles reported on preferences and indicated that not all were using their preferred method.^41^, ^57^, ^63^, ^65^, ^66^ Hopkins et al. reported that while 47% of their sample of college students in Texas preferred short‐acting hormonal methods, only 21% were using these methods. Younger youth had lower odds of using their preferred method in comparison to older youth. Contraception usage also varied across self‐identified race and ethnicities: 34.6% of Hispanics and 55.1% of African Americans were using their primary choice. Long‐acting reversible contraception (LARC) had similar results with 21% of participants preferring LARC but only 9% reporting usage.^65^ For college students, preferred usage if sexual health services were available, and if the campus climate supported contraception and were dependent on where their college was located such as which State in the USA or if the school was in an urban or rural setting.^63^, ^64^, ^65^ Similarly, 13% of surveyed youth from Québec were unable to access their preferred contraceptive, though this study was of lower quality.^57^ Youth often preferred to use methods other than LARC because they did not want a foreign body inside of them and felt a lack of control with LARC.^41^, ^53^, ^63^, ^66^ From a healthcare practitioner standpoint, one study emphasized that LARC should be promoted to youth given its high effectiveness in comparison to other contraceptives.^41^

#### 3.5.2 Youth perceptions and desires for contraception

This theme focused on the social structures making contraception care and knowledge available for youth. Youth also had internal influences that impacted their perspectives and desires for contraception.

##### 3.5.2.1 Contraception approachability

Youth struggled to engage with the services and information required to make contraception choices.^42^, ^59^, ^61^, ^69^, ^70^, ^73^, ^74^ Youth aged 18–21 and members of different racial and ethnic minority communities were comfortable to disclose their contraception needs, ask questions, listen to provider recommendations, and indicated healthcare satisfaction when they had a trusted healthcare provider.^42^, ^59^, ^69^, ^73^ Some mystery callers were provided with either no resources or incorrect information from healthcare workers and staff.^61^, ^74^ Community members' ambivalence towards sexual health education held power over resource availability.^70^

##### 3.5.2.2 Youth perceptions, experiences, and knowledge

Some youth felt contraception was unnatural and they had no need.^71^ Others were concerned that it would “ruin their bodies” and had limited confidence with LARC insertions.^53^, ^73^ There was apprehension around receiving care from pharmacists due to uncertainty of their scope of practice.^54^ Potential reproductive coercion was reported as some were pressured or forced into hormonal contraception by healthcare workers as consent was not always provided.^71^

A total of 12 studies (29%) reported that youth across the USA, Switzerland, and New Zealand had minimal contraception knowledge.^36^, ^39^, ^42^, ^45^, ^46^, ^47^, ^48^, ^50^, ^51^, ^59^, ^63^, ^64^ Youth did not have sexual health education such as types of contraception and where they could access free services; some felt embarrassed by their minimal knowledge.^48^, ^64^ Contraception misconceptions were reported across youth of all ages from different community sizes, and varying levels of educational attainment. Incorrect information included effectiveness, reliability, LARC safety, and IUD eligibility.^41^, ^53^, ^59^, ^66^, ^71^

Concerns or experiences with side effects were discussed in 10 articles (24%).^39^, ^41^, ^46^, ^47^, ^51^, ^58^, ^59^, ^63^, ^66^, ^73^ Side effects included weight gain,^39^, ^45^, ^46^, ^58^, ^59^, ^63^, ^71^ menstruation changes,^45^, ^51^, ^59^, ^71^, ^73^ pain and cramping,^47^, ^59^, ^63^, ^66^, ^71^, ^73^ and mood changes.^39^, ^63^ One study identified LARC side effect concerns among college students, 68% of whom identified as White. Within this group, 28% reported side effect concerns as a barrier to using IUDs and 25% for implants. However, lack of LARC knowledge was the primary reason for lower use, and side effect concerns were only the main reason for not using LARC in 1.9% and 2.6% for IUD and implants, respectively.^63^ Youth were also interested in LARC for non‐contraceptive reasons such as to manage menstruation‐related symptoms, cramps, and acne.^41^, ^66^ Nearly half of articles (46%) primarily focused on the benefits of contraception to prevent pregnancy or sexually transmitted infections.^35^, ^37^, ^38^, ^39^, ^42^, ^43^, ^46^, ^47^, ^49^, ^52^, ^55^, ^56^, ^60^, ^61^, ^62^, ^65^, ^68^, ^71^, ^73^

#### 3.5.3 Youth seeking contraception

This theme identified how social norms and judgments towards youth contraception use affected the ability of youth to look for contraception. Youth identities were also influential in their capacity to pursue contraception.

##### 3.5.3.1 Acceptability of usage

Societal stigma against youth using contraception was described by both youth and nurses^46^, ^56^ and in reference to specific methods of IUDs^53^ and emergency contraception.^47^ There was hesitance about the acceptability of sexual health education for youth as rural community members felt sex education increased youth sexual activity.^70^ This assumption was challenged as another study found young women's “*first‐time contraceptive decision making was not focused on avoiding unintended pregnancy.”* ^41^ Other research outlined the acceptability of programming focused on connecting youth with contraceptive counseling from the perspective of urban youth patients and caregivers.^43^

##### 3.5.3.2 Capacity to pursue contraception

Eight studies (20%) discussed the negative impacts of youth's social identities on their ability to seek care.^35^, ^36^, ^37^, ^42^, ^53^, ^54^, ^69^, ^70^ Specific social identities described included race and ethnicity,^36^, ^69^, ^70^ gender,^36^, ^42^ age,^35^, ^36^, ^54^ geography,^35^, ^37^ income,^35^ and immigration status.^70^ Immigrant and racial or ethnic minority youth reported experiences or fear of discrimination impacting their contraception access.^36^, ^69^, ^70^ American Latina youth who reported perceived racial/ethnic discrimination in every‐day settings had 23% lower odds of contraception service satisfaction in comparison to those without perceived experiences.^69^ Younger youth, those in lower income neighborhoods, and rural communities experienced more barriers finding contraception services than their respective counterparts.^35^, ^36^, ^37^, ^54^ Fears of a pelvic examination and challenging past gynecological medical experiences delayed visits.^40^, ^68^, ^73^

Parents were seen as the most important influencer on youth ability to seek care, and this was true in both high‐ and low‐quality studies. Youth struggled to seek care independently without their accompaniment, consent, and approval often because of their young age and restrictive health or clinic policies, regardless of article quality.^37^, ^38^, ^43^, ^50^, ^54^, ^58^, ^59^, ^60^, ^73^, ^74^, ^75^ Youth partners were also influential in seeking contraception.^42^, ^59^, ^73^

#### 3.5.4 Reaching contraception

This theme showed that the ability to obtain contraception was dependent on availability, care accommodation to youth and overcoming structural barriers.

##### 3.5.4.1 Contraception availability and accommodation to youth

There was minimal access to school‐based programs, availability of trained practitioners, time for private counseling, extended wait times, and minimal sexual health services.^41^, ^47^, ^67^, ^72^, ^73^ Desired methods were not always easily available.^35^, ^37^, ^75^ One study noted, in comparison to urban areas in southwestern USA states, rural national chain pharmacies were 12.4% less likely to have emergency contraception in stock.^37^ Similarly, the odds of pharmacy denial of emergency contraception were 53% higher in rural communities in comparison to urban places.^35^ Health policies limited sexual health services through budget cuts^44^ and service closure during the COVID‐19 pandemic.^60^ Healthcare visits were challenging because of requirements for multiple visits,^45^, ^74^ prescriptions,^37^ age restrictions,^35^, ^50^ and physical examinations.^72^ Counseling was also not prioritized in clinical visits in studies of varying quality.^52^, ^62^

Some practitioners were hesitant to provide LARC because they did not feel confident in placement,^53^ or were reluctant to remove implants.^41^ There was also poor communication among practitioners of who would be discussing contraception with patients^52^ and inadequate care integration among healthcare teams.^45^

Administrative barriers included the difficulty of scheduling a healthcare appointment at a convenient time in both high‐ and low‐quality studies.^41^, ^44^, ^55^, ^60^, ^65^, ^73^, ^74^ There were unclear procedures for scheduling appointments and in some instances, youth were required to share their story several times before reaching care.^37^, ^50^ Contraception services were also reportedly controlled by receptionists who would not make appointments for youth in a lower‐quality study.^60^

##### 3.5.4.2 Structural barriers

Issues with transportation, geography, and limited youth engagement were reported. Both high‐ and low‐quality studies reported challenges for youth to reach care. Transportation challenges were mentioned in five articles.^43^, ^44^, ^50^, ^55^, ^70^ Large travel distances were challenging, particularly for those accessing care without parents knowing and from rural communities.^37^, ^53^ One participant shared “*if your parents don't know you've gotta catch buses and stuff like that, and sometimes that can be a bit hard with appointment times*” (regional focus group 2).^53^ Youth also did not schedule a follow‐up gynecologic appointment,^55^ had poor contraception adherence,^45^, ^46^, ^51^ and self‐censored sexual health needs during the COVID‐19 pandemic.^60^

#### 3.5.5 Contraception costs

The theme of costs associated with contraception was discussed in 16 articles (39%) of various quality.^36,39,41,44,45,53,54,56,57,59‐61,63–65,72^ Costs focused on the direct and associated prices of care and services as well as youth's ability to pay.

##### 3.5.5.1 Contraception affordability

There are many associated costs with contraception.^41^, ^44^, ^54^, ^74^ Younger and older youth from an urban center who primarily identified as African American commented on the high out of pocket fees of contraception and felt cost coverage should be offered to improve access.^54^ Program administrators were also concerned about the high costs and developed sliding‐scale fees or were committed to subsidizing, despite funding cuts.^44^ Youth also described physician reluctance to remove implants because of the higher costs in comparison to placement.^41^ Not all clinics were transparent with costs, only 48% of gynecology practices shared insurance information when asked by mystery‐callers inquiring about IUDs.^74^

##### 3.5.5.2 Youth finances

Youth lacked financial resources and insurance which impeded contraception use in general or for their preferred method.^59^, ^64^, ^65^ Among college students, only 22.9% with no insurance were able to use a desired, more effective method, such as LARC, compared to 47.2% of youth with private insurance.^65^ Youth were sometimes unsure if their insurance covered contraception.^45^, ^54^

Youth's ability to utilize contraception methods and receive quality care was contingent on the joint experiences of accessing care and affordability.

#### 3.5.6 Quality of contraception care

The seventh theme reflects quality of care. including the standard care that youth received, which in turn was influenced by practitioner views about contraception use among youth, confidentiality prioritization, and whether care structures reflected youth needs.

##### 3.5.6.1 Practitioner bias

Youth felt judged and experienced poor treatment by healthcare providers who lacked discretion towards the sensitivity of discussing contraception and could be unhelpful.^36^, ^48^, ^51^, ^61^ Practitioner beliefs and assumptions about youth contraception needs negatively impacted quality‐of‐care, such as if a provider felt someone was too young for LARC.^40^, ^41^, ^61^

##### 3.5.6.2 Confidentiality and care structures

Youth confidentiality and privacy of care concerns were frequently discussed and often focused on fears of parents finding out about their contraception use (39%).^36^, ^38^, ^40^, ^41^, ^43^, ^44^, ^48^, ^49^, ^50^, ^51^, ^54^, ^59^, ^67^, ^70^, ^72^, ^73^ Care invasiveness included being asked too many personal questions, and the experience of IUD insertions and pelvic examinations.^37^, ^66^, ^68^ The importance of shared decision making was emphasized by clinicians and youth to improve quality‐of‐care as this approach focuses on open communication and recognizing youth preferences.^49^, ^69^

#### 3.5.7 Supportive contraception care and informed decisions that reflect youth's contraception needs and choices

Collectively, these previous seven themes affected youth's ability to make contraception decisions and receive supportive and quality care. The final theme identified what supportive contraception care for youth looks like.

Articles commented on the importance of future research that explores the complexities of youth contraceptive decision making and the influences of social determinants of health on contraception access.^36^, ^63^, ^70^ Studies outlined the need to continually evaluate and refine contraceptive programming to ensure high quality and supportive care that reflects youth preferences is provided.^43^, ^51^, ^54^ Individual agency and reproductive autonomy were emphasized as factors needed to make informed reproductive choices.^44^, ^64^ A summary of frequently identified contraception barriers can be found in Table 2.

**TABLE 2:** Summary of contraception barriers experienced by youth in high‐income countries identified in a scoping review of 41 articles.

Category of contraception barrier	Healthcare and social structures and systems (supply)	Youth barriers (demand)
Youth contraception needs and care		Preferred contraception was not available or accessible to youth
Youth perceptions and desires for care	Youth were not comfortable approaching contraception health services and resources	Concerns about the side and long‐term effects of contraception usage
	Minimal support from healthcare workers, staff, and community members about contraception resources	Minimal contraception and sexual health education
		Contraception misconceptions
Youth seeking contraception	Societal stigma of youth using contraception	Youths social identities impacting their ability to look for contraception, such as from experiences of discrimination
		Parental and peer influences impacting youths ability to look for contraception
Reaching contraception	Minimal contraception programming and services for youth.	Geographic and transportation challenges
	Denial of contraception services, and restrictive policies	
	Administrative barriers and challenges of obtaining an appointment	
Contraception costs	High out of pocket fees	Minimal financial resources
Utilizing and quality of contraception methods and care	Youth felt judged by healthcare practitioners and staff	Practitioner beliefs limiting youths contraception choices
		Limited prioritization of confidentiality

## 4 DISCUSSION AND CONCLUSIONS

### 4.1 Summary of findings

This scoping review outlined eight thematic barriers HIC youth experience on their journey to accessing contraception and receiving supportive quality care from both youth and health systems perspectives. Results were consistent across high‐ and low‐quality studies, suggesting consistent presence of these barriers. Contraception resources were not approachable, and youth had minimal knowledge of contraceptive choices. Gatekeeping was reported across several systems of power, including parental control and healthcare settings. Youth needed to overcome several physical barriers, like transportation, and their social identities also affected their experiences trying to access contraception. Youth reported that care confidentiality and invasiveness were concerns and that care quality was influenced by practitioners' biases towards their use of contraception. Addressing these barriers would lead to a more supportive contraception care system.

### 4.2 Comparison with existing literature

The synthesized barriers in this review further contextualize other HIC studies that demonstrate how contraception remains inequitable and inaccessible to youth across HIC. An ecological model focused on adolescent sexual health outlines social influences such as the relationships, communities, societal policies that youth interact with that impact their ability to make reproductive health decisions and access care.^76^ These findings echo and further support the complexities of youth contraception barriers explored in this review.^27^, ^76^ Other studies also highlight the variations, and complexities of contraception access experienced across youth social identities.^77^ Even with universal healthcare coverage, Portuguese youth with lower SES and education levels reported lower contraception use, indicating the continued presence of other contraception barriers outside of cost.^78^ Barriers of minimal contraception knowledge or where to access care, LARC side‐effect and future fertility concerns, stigma, and poor practitioner communication, are clear barriers in other studies.^77^

The importance of youth‐focused care, practitioner training, confidentiality, and strong patient‐practitioner relationships outlined in this review and other research that includes HIC signify priorities of health systems.^16^, ^17^, ^18^ These findings indicate the need and benefits of youth‐focused care; youth‐focused services are recommended to provide appropriate, supportive care. Shared decision making is echoed as a tool to minimize power dynamics in patient‐practitioner relationships.^79^, ^80^ Actions to address contraception barriers like shared decision making are crucial steps to improve care; however, the large youth populations in this review suggest their experiences are not universal. Community engagement is an important consideration to help understand specific barriers, particularly in relation to intersectional experiences that indicate persistent barriers for specific subgroups of youth.

### 4.3 Strengths and limitations

Areas of strength include following the best practices outlined by JBI and PRISMA‐ScR.^20^, ^21^, ^22^ Multiple article reviewers and assessors improved finding consistency. Additional content analysis helped to further capture described barriers.

This review focused on the term “barrier” in general and did not recognize how this term could be defined differently throughout studies. Expanding publication year eligibility may have provided insights on how barriers have shifted over time. Many articles (88%) were in the USA; therefore, findings may be more reflective of American reproductive health policies and youth contraception access. While eight articles (20%) were lower quality according to the MMAT tool, the similarity of results to high quality articles minimizes the risk of poor reliability of described contraception barriers.

### 4.4 Research gaps and areas of future study

Youth age, community size, and SES affected experiences accessing contraception and need to be prioritized in future studies. Few articles discussed the convergence of these identities and how they impacted contraception access and usage. This made it challenging to understand the diversity of experiences across social identities. Gaps in demographics included specifying sex and/or gender, sexual orientation, educational attainment, immigration status, and preferred language. More research regarding sexual and gender minority groups is recommended to better understand contraception use, access, and barriers for these youth populations. We recommend that the intersectionality of youth identities and connections to described barriers be considered in more depth for both quantitative and qualitative research. This will improve understanding of how contraception needs, preferences, interactions with systems of powers, and barriers vary among subpopulations.^28^ Poor MMAT scores for mixed‐methods studies outline the need for stronger research using this design. Quantitative studies were primarily cross‐sectional, and a longitudinal or time‐series study would improve understanding of youth contraception patterns over time.

### 4.5 Implications

Our findings provide perspectives to contraception access and how barriers can impede youth from receiving supportive contraception care. While there was variation in demographics among articles, our findings outline how different social determinants of health influence contraception preferences, needs, and access. This review outlines the importance of accessible contraception and counseling that respect and support youths' autonomy, and preferences, so they can make informed reproductive choices. Identifying barriers through this review provides an opportunity for health policy makers, sexual health community members and clinicians to improve access to contraception for youth; these efforts will be influential to reaching the UNs goal of universal contraception access by 2030.^1^

## AUTHOR CONTRIBUTIONS

All authors have fulfilled ICMJE authorship criteria. BKJ, conceptualized the project, developed the protocol, executed the search criteria, data analysis, article quality assessment, and manuscript writing and revisions. PJ assisted with protocol development, and manuscript revisions. MO, CP, and ZK assisted with article screening and provided manuscript revisions. PSF and CM helped to complete article quality assessments and provided manuscript revisions. CM also reviewed article citations and tables for consistency. As doctoral committee members, SM and LS assisted with supervision and provided project design, analyses, and manuscript writing and revisions. KM supervised this work and assisted with protocol refinement, oversaw data collection, study analyses, and manuscript writing and revisions. All authors have provided final approval of the manuscript and agree to be accountable for all aspects of the work.

## FUNDING INFORMATION

This project received no funding to conduct this research. BKJ is supported by a Doctoral Research Award from the Canadian Institute of Health Research. Her doctoral work was also supported from 2023 to 2024 by a Graduate and Fellowship Research Award in Women's Health from the Women's Health Research Institute at BC Women's Hospital.

## CONFLICT OF INTEREST STATEMENT

The authors have no conflicts of interest to declare associated with this scoping review.

## Supporting information


**Data S1.** (PRISMA‐ScR) Checklist.

**Data S2.** Barriers to Contraception Access and Use Among Youth: A Scoping Review in High‐Income Countries Supplemental 2: Search Criteria.

**Table S1.** List of Included in this Scoping Review on Youth Contraception Barriers in High Income Countries (*n* = 41).

**Table S2A.** Mixed Methods Appraisal Tool Version 2018 Article Assessments for Qualitative and Quantitative Non‐Randomized Articles in this Scoping Review on Youth Contraception Barriers in High Income Countries (*n* = 41). Consensus reached between two assessors. Only Relevant Study Designs Pertaining to this Review are Included.
**Table S2B.** Mixed Methods Appraisal Tool Version 2018 Article Assessments of Quantitative Descriptive and Mixed‐Methods Articles in this Scoping Review on Youth Contraception Barriers in High Income Countries (*n* = 41). Consensus reached between two assessors. Only Relevant Study Designs Pertaining to this Review are Included.

**Table S3.** List of Included Demographics in this Scoping Review on Youth Contraception Barriers in High Income Countries (*n* = 41).

## Data Availability

Data sharing is not applicable to this article as no new data were created or analyzed in this study.
