# Supplementary material for: Barriers to contraception access and use among youth: A scoping review in high‐income countries
Source: Int J Gynaecol Obstet. 2025 Nov 14;173(1):74–86. doi: 10.1002/ijgo.70637 (PMC12988402; doi:10.1002/ijgo.70637)
Supplement: Supplementary file 2 — Data S2. Barriers to Contraception Access and Use Among Youth: A Scoping Review in High‐Income Countries Supplemental 2: Search Criteria. [file IJGO-173-74-s002.docx]

**Barriers to Contraception Access and Use Among Youth: A Scoping Review in High-Income Countries Supplemental 2: Search Criteria**

Search criteria across the MEDLINE, EMBASE, and CINAHL databases.

| **#** | **DATABASE: MEDINE** |
| --- | --- |
| 1 | ((unmet or barrier) adj3 contracept*).ti,ab,kf. [unmet/barrier contraception] |
| 2 | ((unmet or barrier) adj3 family planning).ti,ab,kf. [unmet/barrier family planning] |
| 3 | ((unmet or barrier) adj3 (LARC or Long Acting Reversible Contracept*)).ti,ab,kf. [unmet/barrier LARC] |
| 4 | ((unmet or barrier) adj3 Birth Control).ti,ab,kf. [unmet/barrier Birth Control] |
| 5 | 1 or 2 or 3 or 4 [combined unmet/ barrier contraceptives] |
| 6 | (youth or high school student* or universit* student* or young adult* or emerging adult* or young people or teen* or adolescen*).ti,ab,kf. [Youth terms] |
| 7 | exp Adolescent/ or exp young adult/ [Youth MeSH] |
| 8 | 6 or 7 [youth combined] |
| 9 | 5 and 8 [unmet/barrier contraception + youth] |
| 10 | limit 9 to yr="2013 - Current" [10 year limit] OR  limit 9 to dt=20230918-20240921 [subsequent search] |
| 11 | (exp africa/ or exp caribbean region/ or exp central america/ or latin america/ or mexico/ or exp south america/ or exp developing country/ or exp Asia/ or exp Middle East/) not Japan.mp. [mp=title, book title, abstract, original title, name of substance word, subject heading word, floating sub-heading word, keyword heading word, organism supplementary concept word, protocol supplementary concept word, rare disease supplementary concept word, unique identifier, synonyms, population supplementary concept word, anatomy supplementary concept word] |
| 12 | ((low and middle income countr*) or LMIC).ti,ab,kf. [LMIC terms] |
| 13 | 11 or 12 [LMIC combined] |
| 14 | 10 not 13 [unmet contraception + youth, eliminate LMIC, last 10 years] |
| 15 | (contracept* or condom* or birth control or family planning or IUD or Long Acting Reversible Contracept*).ti,ab,kf. [contraceptives terms] |
| 16 | exp contraception/ or exp contraception, barrier/ or exp hormonal contraception/ or exp long-acting reversible contraception/ or exp Contraceptive Devices/ or exp Contraceptive Agents/ [contraception MeSH terms] |
| 17 | 15 or 16 [combined contraceptives] |
| 18 | (access* adj3 (service* or care* or contracept* or health* or family planning or birth control or IUD or Long Acting Reversible Contracept*)).ti,ab,kf. [contraception access] |
| 19 | (uptake* adj3 (service* or care* or contracept* or health* or family planning or birth control or IUD or Long Acting Reversible Contracept*)).ti,ab,kf. [contraception uptake] |
| 20 | (accept* adj3 (service* or care* or contracept* or health* or family planning or birth control or IUD or Long Acting Reversible Contracept*)).ti,ab,kf. [contraception acceptable] |
| 21 | (barrier* adj3 (service* or care* or contracept* or health* or family planning or birth control or IUD or Long Acting Reversible Contracept*)).ti,ab,kf. [contraception barrier] |
| 22 | 18 or 19 or 20 or 21 [combine contraception access, uptake, acceptability, barrier] |
| 23 | (unmet or barrier* or poor or inaccessible*).ti,ab,kf. [inaccessible and barrier] |
| 24 | (8 and 17 and 22 and 23) not 13 [youth and combine contraception access, uptake and acceptability, and contraceptives exclude LMIC] |
| 25 | limit 24 to dt=20230918-20240921 [youth and combine contraception access, uptake and acceptability, and contraceptives exclude LMIC, September 18 2023-September 21 2024] |
| 26 | 14 and 25 [combine both searches -papers that overlap] |
| 27 | 14 or 25 [combine both searches, apparent from and there is not much overlap] |
| 28 | limit 27 to yr="2013 - Current" [youth and combine contraception access, uptake and acceptability, and contraceptives exclude LMIC, last 10 years] OR  limit 27 to yr="2023 - 2024" |

| **#** | **DATABASE: EMBASE** |
| --- | --- |
| 1 | ((unmet or barrier) adj3 contracept*).ti,ab,kf. [unmet/barrier contraception] |
| 2 | ((unmet or barrier) adj3 family planning).ti,ab,kf. [unmet/barrier family planning] |
| 3 | ((unmet or barrier) adj3 (LARC or Long Acting Reversible Contracept*)).ti,ab,kf. [unmet/barrier LARC] |
| 4 | ((unmet or barrier) adj3 Birth Control).ti,ab,kf. [unmet/barrier Birth Control] |
| 5 | 1 or 2 or 3 or 4 [combined unmet/ barrier contraceptives] |
| 6 | (youth or high school student* or universit* student* or young adult* or emerging adult* or young people or teen* or adolescen*).ti,ab,kf. [Youth terms] |
| 7 | exp Juvenile/ or exp Adolescent/ or exp Young adult/ or exp Student/ [Youth MeSH] |
| 8 | 6 or 7 [youth combined] |
| 9 | 5 and 8 [unmet/barrier contraception + youth] |
| 10 | limit 9 to yr="2013 - Current" [10 year limit] OR  limit 9 to dd="20230918-20240921" [re run search] |
| 11 | (exp africa/ or exp caribbean region/ or exp central america/ or latin america/ or mexico/ or exp south america/ or exp developing country/ or exp Low income country/ or exp Asia/ or exp Middle East/) not Japan.mp. [mp=title, abstract, heading word, drug trade name, original title, device manufacturer, drug manufacturer, device trade name, keyword heading word, floating subheading word, candidate term word] |
| 12 | ((low and middle income countr*) or LMIC).ti,ab,kf. [LMIC terms] |
| 13 | 11 or 12 [LMIC combined] |
| 14 | 10 not 13 [unmet contraception + youth, eliminate LMIC, last 10 years] |
| 15 | (contracept* or condom* or birth control or family planning or IUD or Long Acting Reversible Contracept*).ti,ab,kf. [contraceptives terms] |
| 16 | exp contraception/ or exp long-acting reversible contraception/ or exp Contraceptive Agents/ or exp Family Planning/ or exp Intrauterine Contraceptive Device/ or exp Condom/ or exp Birth Control/ [contraception MeSH terms] |
| 17 | 15 or 16 [combined contraceptives] |
| 18 | (access* adj3 (service* or care* or contracept* or health* or family planning or birth control or IUD or Long Acting Reversible Contracept*)).ti,ab,kf. [contraception access] |
| 19 | (uptake* adj3 (service* or care* or contracept* or health* or family planning or birth control or IUD or Long Acting Reversible Contracept*)).ti,ab,kf. [contraception uptake] |
| 20 | (accept* adj3 (service* or care* or contracept* or health* or family planning or birth control or IUD or Long Acting Reversible Contracept*)).ti,ab,kf. [contraception acceptable] |
| 21 | (barrier* adj3 (service* or care* or contracept* or health* or family planning or birth control or IUD or Long Acting Reversible Contracept*)).ti,ab,kf. [contraception barrier] |
| 22 | 18 or 19 or 20 or 21 [combine contraception access, uptake, acceptability, barrier] |
| 23 | (unmet or barrier* or poor or inaccessible*).ti,ab,kf. [inaccessible and barrier] |
| 24 | Unmet medical need/ or Healthcare access/ or Health care need.mp. [mp=title, abstract, heading word, drug trade name, original title, device manufacturer, drug manufacturer, device trade name, keyword heading word, floating subheading word, candidate term word] |
| 25 | (8 and 17 and 22 and 23 and 24) not 13 [youth and combine contraception access, uptake and acceptability, and contraceptives exclude LMIC] |
| 26 | limit 25 to dd="20230918-20240921" [youth and combine contraception access, uptake and acceptability, and contraceptives exclude LMIC, September 18 2023-September 21 2024]  OR  limit 25 to yr="2013 - Current" [youth and combine contraception access, uptake and acceptability, and contraceptives exclude LMIC, last 10 years] |
| 27 | 14 and 26 [combine both searches -papers that overlap] |
| 28 | 14 or 26 [combine both searches, apparent from and there is not much overlap] |

| **Query** | **DATABASE: CINAHL** | |
| --- | --- | --- |
| S20 | S13 OR S18 | Limiters - Publication Date: 20130101-20231231 Search modes - Proximity |
| S19 | S13 AND S18 | Search modes - Proximity |
| S18 | S5 AND S17 NOT S12 | Limiters - Publication Date: 20130101-20231231 Search modes - Proximity |
| S17 | S14 AND S15 AND S16 | Limiters - Publication Date: 20130101-20231231 Search modes - Proximity |
| S16 | unmet needs or challenges or impact or barriers | Limiters - Publication Date: 20130101-20231231 Search modes - Proximity |
| S15 | (MH "Health Services Accessibility+") OR (MH "Access to Primary Care") OR (MH "Health Services Needs and Demand+") OR "access to care or access to healthcare or access to services" OR ( uptake or participation or motivations or barriers to uptake or adherence ) OR ( acceptance or attitudes or perception ) | Limiters - Publication Date: 20130101-20231231 Search modes - Proximity |
| S14 | ( (MH "Contraceptives, Oral Combined") OR (MH "Contraceptives, Oral+") OR (MH "Contraception+") OR (MH "Contraceptives, Postcoital+") OR (MH "Family Planning+") OR (MH "Contraceptive Agents, Male") OR (MH "Contraceptive Agents+") OR (MH "Hormonal Contraception") OR (MH "Contraceptive Devices+") OR (MH "Contraceptive Agents, Hormonal+") OR (MH "Long-Acting Reversible Contraceptives") OR (MH "Intrauterine Devices") OR "contraception or birth control or family planning or contraceptive or pregnancy prevention" ) OR ( (MH "Long-Acting Reversible Contraceptives") or larc or long acting reversible contraception ) | Limiters - Publication Date: 20130101-20231231 Search modes - Proximity |
| S13 | #s6 NOT #s12 | Search modes - Proximity |
| S12 | S7 OR S8 OR S9 OR S10 OR S11 | Limiters - Publication Date: 20130101-20231231 Search modes - Proximity |
| S11 | (MH "Asia+") OR (MH "Asia, Southern+") OR (MH "Asia, Western+") OR (MH "Asia, Southeastern+") OR (MH "Asia, Central+") OR (MH "Vietnam") OR (MH "Uzbekistan") OR (MH "United Arab Emirates") OR (MH "Turkmenistan") OR (MH "Tibet") OR (MH "Thailand") OR (MH "Tajikistan") OR (MH "Sri Lanka") OR (MH "Southeast Asians+") OR (MH "South Asians") OR (MH "Singapore") OR (MH "Philippines") OR (MH "Pakistan") OR (MH "Nepal") OR (MH "Malaysia") OR (MH "Kyrgyzstan") OR (MH "Kazakhstan") OR (MH "Indonesia") OR (MH "India") OR (MH "China+") OR (MH "Cambodia") OR (MH "Bhutan") OR (MH "Bangladesh") OR (MH "Azerbaijan") OR (MH "Afghanistan") | Limiters - Publication Date: 20130101-20231231 Search modes - Proximity |
| S10 | (MH "Latin America") OR (MH "South America+") OR (MH "Central America+") | Limiters - Publication Date: 20130101-20231231 Search modes - Proximity |
| S9 | (MH "West Indies+") OR (MH "Virgin Islands of the United States") OR (MH "Cuba") OR "caribbean" | Limiters - Publication Date: 20130101-20231231 Search modes - Proximity |
| S8 | (MH "Low and Middle Income Countries") OR (MH "Developing Countries") | Limiters - Publication Date: 20130101-20231231 Search modes - Proximity |
| S7 | (MH "Africa+") OR (MH "Africa South of the Sahara+") OR (MH "Africa, Western+") OR (MH "Africa, Central+") OR (MH "Africa, Southern+") OR (MH "Africa, Eastern+") OR (MH "Africa, Northern+") OR (MH "Namibia") OR (MH "Zimbabwe") OR (MH "Zambia") OR (MH "Yohimbe") OR (MH "West Africans") OR (MH "Uganda") OR (MH "Tunisia") OR (MH "Togo") OR (MH "Tanzania") OR (MH "Swaziland") OR (MH "Sudan") OR (MH "Sub-Saharan Africans+") OR (MH "Southern Africans") OR (MH "Somalia") OR (MH "Sierra Leone") OR (MH "Senegal") OR (MH "Niger") OR (MH "Nigeria") OR (MH "Mozambique") OR (MH "Morocco") OR (MH "Middle East+") OR (MH "Mauritania") OR (MH "Mali") OR (MH "Malawi") OR (MH "Madagascar") OR (MH "Guinea-Bissau") OR (MH "Guinea") OR (MH "Ghana") OR (MH "Gambia") OR (MH "Gabon") OR (MH "Ethiopia") OR (MH "Eritrea") OR (MH "Equatorial Guinea") OR (MH "Egypt") OR (MH "East Africans") OR (MH "Djibouti") OR (MH "Democratic Republic of the Congo") OR (MH "Chad") OR (MH "Central Africans") OR (MH "Central African Republic") OR (MH "Cape Verde") OR (MH "Cameroon") OR (MH "Burundi") OR (MH "Burkina Faso") OR (MH "Botswana") OR (MH "Benin") OR (MH "Arabs") OR (MH "Angola") OR (MH "Algeria") | Limiters - Publication Date: 20130101-20231231 Search modes - Proximity |
| S6 | S4 AND S5 | Search modes - Proximity |
| S5 | (MH "Young Adult") OR (MH "Adolescence+") OR "youth or adolescents OR (young people or teen or young adults) OR (( high school students or teenagers or secondary schools or adolescents ) OR ( college students or university students or undergraduates )) | Limiters - Publication Date: 20130101-20231231 Search modes - Proximity |
| S4 | S1 OR S2 OR S3 | Limiters - Publication Date: 20130101-20231231 Search modes - Proximity |
| S3 | (MH "Contraceptives, Oral Combined") OR (MH "Contraceptives, Oral+") OR (MH "Contraception+") OR (MH "Contraceptives, Postcoital+") OR (MH "Family Planning+") OR (MH "Contraceptive Agents, Male") OR (MH "Contraceptive Agents+") OR (MH "Hormonal Contraception") OR (MH "Contraceptive Devices+") OR (MH "Contraceptive Agents, Hormonal+") OR (MH "Long-Acting Reversible Contraceptives") OR (MH "Intrauterine Devices") OR "contraception or birth control or family planning or contraceptive or pregnancy prevention" AND "unmet needs or challenges or impact or barriers" | Limiters - Publication Date: 20130101-20231231 Search modes - Proximity |
| S2 | (MH "Long-Acting Reversible Contraceptives") or larc or long acting reversible contraception AND "unmet needs or challenges or impact or barriers" | Limiters - Publication Date: 20130101-20231231 Search modes - Proximity |
| S1 | ( contraception or birth control or family planning or contraceptive or pregnancy prevention ) AND "unmet needs or challenges or impact or barriers" | Limiters - Publication Date: 20130101-20231231 Search modes - Proximity |
