# Supplementary material for: Barriers to contraception access and use among youth: A scoping review in high‐income countries
Source: Int J Gynaecol Obstet. 2025 Nov 14;173(1):74–86. doi: 10.1002/ijgo.70637 (PMC12988402; doi:10.1002/ijgo.70637)
Supplement: Supplementary file 3 — Table S1. List of Included in this Scoping Review on Youth Contraception Barriers in High Income Countries (n = 41). [file IJGO-173-74-s003.docx]

| **Supplemental Table 1 (Table S1).** List of Included in this Scoping Review on Youth Contraception Barriers in High Income Countries (n=41) | | | | | | | |
| --- | --- | --- | --- | --- | --- | --- | --- |
| **Article Title** | **Article Citation** | **Publication Year** | **Study Location** | **Study Objective(s)/ Question(s)** | **Study Type** | **Study Methods** | **Participants** |
| "I don't know enough to feel comfortable using them:" Women's knowledge of and perceived barriers to long-acting reversible contraceptives on a college campus | Hall KS, Ela E, Zochowski MK, Caldwell A, Moniz M, McAndrew L, Steel M, Challa S, Dalton VK, Ernst S. “I don't know enough to feel comfortable using them:” Women's knowledge of and perceived barriers to long-acting reversible contraceptives on a college campus. Contraception. 2016 Jun 1;93(6):556-64. | 2016 | The United States of America (Michigan). | *“To assess multiple dimensions of long-acting reversible contraception (LARC) knowledge and perceived multi-level barriers to LARC use among a sample of college women.”* | Quantitative | **Data Collection:** Quantitative Survey.  **Data Analysis:** Descriptive statistics, bivariate tests, multivariable linear regression. | Undergraduate female students at an American University (aged 18- ≥22, n=1982). |
| "It seems kinda like a different language to us": Homeless youths' attitudes and experiences pertaining to condoms and contraceptives | Begun S, Combs KM, Torrie M, Bender K. “It seems kinda like a different language to us”: Homeless youths’ attitudes and experiences pertaining to condoms and contraceptives. Social work in health care. 2019 Mar 16;58(3):237-57. | 2019 | The United States of America (Colorado). | *“Homeless youths’ attitudes and lived experiences pertaining to condom and other contraceptive use. The*  *broader “phenomenon” examined was experiencing homelessness as a young person while grappling with choices regarding reproductive and sexual health.”* | Qualitative | **Data Collection:**  Semi-structured interviews.  **Data Analysis**: Iterative open,  holistic, and focused coding. | Youth at an overnight youth shelter (aged 18-21, n=30). |
| "The thing in my arm": Providing contraceptive services for adolescents in primary care | Lewin A, Skracic I, Brown E, Roy K. “The thing in my arm”: Providing contraceptive services for adolescents in primary care. Women's Health. 2024 May;20:17455057241248399. | 2024 | The United States of America (Delaware). | *“Assess the practice-level barriers and facilitators to providing contraceptive care, particularly long-acting reversible contraceptives (LARCs), to adolescents in primary care settings.”* | Qualitative | **Data Collection:** In-depth, semi-structured interviews.  **Data Analysis:** Grounded theory approach (open, axial, and selective coding). | Leaders (administrators or clinician administrators) from DelCAN primary care sites (n=16). |
| A mystery shopper study identifying practice-level barriers to adolescent IUD access in western Pennsylvania | Lim SE, Krajewski CM. A mystery shopper study identifying practice-level barriers to adolescent IUD access in western Pennsylvania. Contraception. 2020 Feb 1;101(2):130-1. | 2020 | The United States of America (Pennsylvania). | *“To identify practice-level barriers that adolescents experience when seeking a hormonal intrauterine device (IUD) using a mystery shopper approach”* | Quantitative | **Data Collection:** Mystery callers stated they were “*nulliparous*  *sixteen-year-old female seeking information about obtaining*  *a hormonal intrauterine device.”*  **Data Analysis**: Not entirely clear but looks like descriptive statistics. | 56 Obstetrics and Gynecology Clinics contacted. A mix of healthcare workers (nurses, nurse practitioners, medical assistants).  Mystery caller stated they were “*a nulliparous, sixteen-year-old female seeking information about obtaining*  *a hormonal intrauterine device.”* |
| A Pediatric Emergency Department Intervention to Increase Contraception Initiation Among Adolescents | Hoehn EF, Hoefgen H, Chernick LS, Dyas J, Krantz L, Zhang N, Reed JL. A pediatric emergency department intervention to increase contraception initiation among adolescents. Academic Emergency Medicine. 2019 Jul;26(7):761-9. | 2019 | The United States of America. | *“The objectives of this PED-based pilot intervention study were to (1) assess the rate of contraception initiation after contraceptive counseling and appointment facilitation in the PED during the study period (2) identify barriers to successful contraception initiation and (3) determine adolescent acceptability of the intervention.”* | Quantitative | **Data Collection:** pre and post intervention surveys. Electronic medical record review. (Quantitative analysis of an intervention).  **Data Analysis:** Descriptive statistics, Chi-Square or Fisher’s exact tests, odds ratio calculations, and analysis of variances (ANOVA). | Females aged 14–19 at risk for  unintended pregnancy at an urban Level 1 Pediatric emergency department (n=100). |
| A Qualitative Analysis of Long-Acting Reversible Contraception | Sundstrom B, Baker-Whitcomb A, DeMaria AL. A qualitative analysis of long-acting reversible contraception. Maternal and Child Health Journal. 2015 Jul;19:1507-14. | 2015 | The United States of America (Southeast region). | *“How do female college students*  *living in the United States perceive long-acting reversible*  *contraceptive options?* | Qualitative | **Data Collection:**  In-depth interviews.  **Data Analysis:**  Grounded theory approach developed by  Glaser and Strauss. | Women aged 18-24 (n=53) |
| A Qualitative Assessment to Understand the Barriers and Enablers Affecting Contraceptive Use Among Adolescent Male Emergency Department Patients | Chernick LS, Siden JY, Bell DL, Dayan PS. A qualitative assessment to understand the barriers and enablers affecting contraceptive use among adolescent male emergency department patients. American journal of men's health. 2019 Feb;13(1):1557988319825919. | 2019 | The United States of America. | *“To identify the barriers and enablers affecting contraceptive and condom use among adolescent male [Emergency Department] ED patients.”* | Qualitative | **Data Collection:**  Semi-structured interviews.  **Data Analysis:**  Thematic analysis, theoretically approached with the Social Ecological Model. | Males aged 14-19 in an ED for those who were ever sexually active with females (n=24). |
| A Qualitative Study Exploring Contraceptive Practices and Barriers to Long-Acting Reversible Contraceptive Use in a Sample of Adolescents Living in the Southern United States | Coates C, Gordon CM, Simpson T. A qualitative study exploring contraceptive practices and barriers to long-acting reversible contraceptive use in a sample of adolescents living in the Southern United States. Journal of Pediatric and Adolescent Gynecology. 2018 Dec 1;31(6):605-9. | 2018 | The United States of America (Southern). | *“To understand contraceptive practices of female adolescents in the Deep South and determine barriers to their use of long-acting reversible contraception (LARC).”* | Qualitative | **Data Collection:**  Semi-structured interviews.  **Data Analysis:**  Content analysis. | Sexually active girls aged 14 and 21 years not currently using LARC (n=15). |
| A State-Level Examination of School Nurses' Perceptions of Condom Availability Accompanied by Sex Education | Smith S, Platt JM, Clifford D, Preston M, Satterwhite C, Kelly PJ, Ramaswamy M. A State-Level Examination of School Nurses’ Perceptions of Condom Availability Accompanied by Sex Education. The Journal of School Nursing. 2020 Oct;36(5):386-93. | 2020 | The United States of America (Kansas). | *“Assess school nurse’s perceptions of and comfort level with sex education programs that make condom available in high schools in a state with an abstinence-only program.”* | Mixed-Methods | **Data Collection:**  Quantitative and qualitative online survey.  **Data Analysis:** Univariate and bivariate statistical analyses, grounded theory approach and “*a series of a priori codes were developed to aid in*  *the iterative analysis.”* | High school nurses in Kansas (n=87) |
| Access to Emergency Contraception After Removal of Age Restrictions | Wilkinson TA, Clark P, Rafie S, Carroll AE, Miller E. Access to emergency contraception after removal of age restrictions. Pediatrics. 2017 Jul 1;140(1). | 2017 | The United States of America (Tennessee, Pennsylvania, Ohio, Texas, and Oregon). | “*Starting in 2013, the US Food and Drug Administration removed age*  *restrictions, enabling EC to be sold over the counter to all consumers. We sought to compare*  *the availability and access for female adolescents with the 2012 study, using the same study*  *design.”* | Quantitative | **Data Collection:**  Mystery caller approach where transcripts were coded with respect to different selected variables.  **Data Analysis:**  Descriptive statistics, logistic regression (addition of interaction term between study year and neighbourhood income to compare the two studies). | Pharmacies in 5 cities across The United States of America (n=979). Spoke to pharmacy staff.  Mystery Callers posed as “*17-year-old adolescents in need of [emergency contraception].”* |
| Access to Reproductive Health Care in Juvenile Justice Facilities | Suresh SC, Questell L, Sufrin C. Access to reproductive health care in juvenile justice facilities. Journal of pediatric and adolescent gynecology. 2020 Jun 1;33(3):296-301. | 2020 | The United States of America. | *“Identify access to contraceptive counseling and methods for young women in the juvenile justice system.”* | Quantitative | **Data Collection:**  Cross-sectional survey with quantitative and qualitative questions.  **Data Analysis:**  Descriptive statistics. | State-level health care administrators in juvenile justice systems (n=21) |
| Adolescents' and Young Adults' Reports of Barriers to Confidential Health Care and Receipt of Contraceptive Services | Fuentes L, Ingerick M, Jones R, Lindberg L. Adolescents' and young adults' reports of barriers to confidential health care and receipt of contraceptive services. Journal of Adolescent Health. 2018 Jan 1;62(1):36-43. | 2018 | The United States of America. | *“To describe adolescents’ and young adults’ concerns about confidential reproductive health care and experience with time alone with a provider and examine the association of these confidentiality issues with receipt of contraceptive services.”* | Quantitative | **Data Collection:**  2013-2015 National Survey of Family Growth.  **Data Analysis:**  Descriptive statistics, prevalence calculations, risk ratios (unadjusted and adjusted). | American females aged 15-25 (n=2325). |
| Adolescents' perceived barriers to accessing sexual and reproductive health services in California: a cross-sectional survey | Decker MJ, Atyam TV, Zárate CG, Bayer AM, Bautista C, Saphir M. Adolescents’ perceived barriers to accessing sexual and reproductive health services in California: a cross-sectional survey. BMC Health Services Research. 2021 Dec;21:1-2. | 2021 | The United States of America (California). | *“Quantitatively assess adolescents’ perceptions of barriers to accessing SRH  services by demographic and behavioral characteristics prior to receiving sexual health education”* | Quantitative | **Data Collection:**  Survey  **Data Analysis:**  Descriptive statistics, one-way ANOVA with Bonferroni adjustments for multiple comparisons, logistic regression. | Youth aged 10-19 participants in California’s Personal Responsibility  Education Program (n=10015). |
| Assessing perspectives on an intervention connecting adolescents in outpatient psychiatry care to contraceptive counseling in the United States | Underwood AL, Hyzak KA, Ebersole A, Bunger AC, Berlan ED. Assessing perspectives on an intervention connecting adolescents in outpatient psychiatry care to contraceptive counseling in the United States. Perspectives on Sexual and Reproductive Health. 2024 Mar 27. | 2024 | The United States of America (Midwestern State). | *“To advance the design and tailoring of Link2BC to close gaps in access to SRH care for adolescents with psychiatric disorders.”* | Qualitative | **Data Collection:**  Semi-structured group interviews. (Qualitative assessment of an intervention).  **Data Analysis:**  Content analysis. | Adolescents and their caregivers (psychiatrists, advance practice providers, and registered nurses) at a psychiatry visit in a pediatric hospital (n= 7 youth, n=9 caregivers). |
| Availability and Accessibility of Emergency Contraception to Adolescent Callers in Pharmacies in Four Southwestern States | Uysal J, Tavrow P, Hsu R, Alterman A. Availability and accessibility of emergency contraception to adolescent callers in pharmacies in four southwestern states. Journal of Adolescent Health. 2019 Feb 1;64(2):219-25. | 2019 | The United States of America (Arizona, California, New  Mexico, and Utah). | *“To evaluate the availability and accessibility of emergency contraception (EC) to adolescents in U.S. pharmacies across four Southwestern states, 3 years after the federal Food and Drug Administration (FDA) removed age restrictions for over-the-counter sales of levonorgestrel-only pills.”* | Quantitative | **Data Collection:**  Mystery callers posed as 16-year-olds to pharmacies, callers completed a survey on their experiences.  2015 census data for ZIP codes.  **Data Analysis:**  Descriptive statistics, Pearson Chi-Square, ANOVA, urban and rural ZIP code comparisons, summary variable. | Randomly selected pharmacies across 4 states (n=1127). Pharmacists and pharmacy staff.  Mystery callers posed as “*16-year-olds who wanted to prevent a pregnancy after recent unprotected sex.”* |
| Availability of Confidential Services for Teens Declined After the 2011–2013 Changes to Publicly Funded Family Planning Programs in Texas | Coleman-Minahan K, Hopkins K, White K. Availability of confidential services for teens declined after the 2011–2013 changes to publicly funded family planning programs in Texas. Journal of Adolescent Health. 2020 Jun 1;66(6):719-24. | 2020 | The United States of America (Texas). | *“Texas is one of 24 states that does not explicitly allow minors to consent to contraception. We explore changes in the provision of confidential reproductive health services after the implementation of state policies that*  *cut and reorganized public family planning funding,*  *including Title X.”* | Qualitative | **Data Collection:**  In-depth interviews.  **Data Analysis:**  Thematic analysis. | Program administrators at publicaly funded family planning groups in Texas (n=47). |
| Barriers to adolescent contraception use and adherence | Clare C, Squire MB, Alvarez K, Meisler J, Fraser C. Barriers to adolescent contraception use and adherence. International journal of adolescent medicine and health. 2016 Oct 15;30(4):20160098. | 2016 | The United States of America. | *“To determine if there are additional barriers to contraception use and adherence among  an ethnically diverse urban population.”* | Quantitative | **Data Collection:**  Survey  **Data Analysis:**  Descriptive statistics, Chi-Squared, phi-coefficient, Contingency Coefficient, Cramer’s V, and Satterthwaite. | Female patients aged 13-21 at a Metropolitan Hospital Center (n=63). |
| Barriers to and enablers of contraceptive use among adolescent females and their interest in an emergency department based intervention | Chernick LS, Schnall R, Higgins T, Stockwell MS, Castaño PM, Santelli J, Dayan PS. Barriers to and enablers of contraceptive use among adolescent females and their interest in an emergency department based intervention. Contraception. 2015 Mar 1;91(3):217-25. | 2015 | The United States of America. | *“To identify the barriers to and enablers*  *of contraceptive use among adolescent females using the ED and determine their interest in an ED-based pregnancy prevention intervention.”* | Qualitative | **Data Collection:**  Semi-structured interviews. (Qualitative assessment of an intervention).  **Data Analysis:**  General qualitative approach. Modified the Health Belief Model for ode organization. | Sexually active females aged 14–19 presenting with reproductive health complaints and at risk for pregnancy (contraception non-use) in the emergency department (n=14). |
| Barriers to Long-Acting Reversible Contraceptive Uptake Among Homeless Young Women | Dasari M, Borrero S, Akers AY, Sucato GS, Dick R, Hicks A, Miller E. Barriers to long-acting reversible contraceptive uptake among homeless young women. Journal of pediatric and adolescent gynecology. 2016 Apr 1;29(2):104-10. | 2016 | The United States of America (Pennsylvania). | *“To identify barriers to long-acting reversible contraception (LARC) uptake among homeless young women.”* | Mixed-Methods | **Data Collection:**  Survey and interviews  **Data Analysis:**  Descriptive statistics and directive content analysis. | Women aged 18-24 with a past year history of homelessness (n=15). |
| Can youth get the contraception they want? Results of a pilot study in the province of Quebec | Di Meglio G, Yeates J, Seidman G. Can youth get the contraception they want? Results of a pilot study in the province of Quebec. Paediatrics & Child Health. 2020 Apr 10;25(3):160-5. | 2020 | Canada (Quebec). | *“Understanding Canadian youths’ contraceptive*  *experience with specific attention to the systemic*  *barriers that prevent them from initiating or continuing a*  *desired method.”* | Mixed Methods | **Data Collection:**  Survey with quantitative and qualitative questions. Geomapping of postal codes.  **Data Analyses:**  Descriptive statistics, Chi-Square, and Batch Geo. Bertrand’s classification for qualitative open-responses. | Quebec youth aged 14 to 21(n=105). |
| Concerns About the Cost of Contraception Among Young Women Attending Community College | Yarger J, Schroeder R, Blum M, Cabral MA, Brindis CD, Perelli B, Harper CC. Concerns About the Cost of Contraception Among Young Women Attending Community College. Women's Health Issues. 2021 Sep 1;31(5):420-5. | 2021 | The United States of America (California and Oregon). | *“Assessed concerns about the cost of contraception among young women aged 18–25 years who were attending community college in California and Oregon. We examined variation in cost concerns by women’s health insurance coverage and access to publicly funded family planning services.”* | Quantitative | **Data Collection:**  Surveys (pre and post quantitative assessment of an educational intervention).  **Data Analysis:**  Descriptive statistics, Chi-Square test, and mixed-effects logistic regression. | Students from 5 community colleges aged 18-25 who self-identified as *“female, had vaginal sex, and were not pregnant or trying to become pregnant”* (n=389). |
| Delayed Visits for Contraception Due to Concerns Regarding Pelvic Examination Among Women with History of Intimate Partner Violence | Holt HK, Sawaya GF, El Ayadi AM, Henderson JT, Rocca CH, Westhoff CL, Harper CC. Delayed visits for contraception due to concerns regarding pelvic examination among women with history of intimate partner violence. Journal of general internal medicine. 2021 Jul;36:1883-9. | 2021 | The United States of America) | *“We examined women’s attitudes towards*  *pelvic examination with history of intimate partner violence*  *(pressured to have sex, or verbal, or physical abuse).”* | Quantitative | **Data Collection:**  Randomised controlled trial baseline survey.  **Data Analysis:** Descriptive statistics, *Pearson’s* Chi-Squared testing, multivariable generalized estimating  equations with a logit link. | Women aged 18-25 from 40 reproductive health centres (n=1490). |
| Disrupted prevention: condom and contraception access and use among young adults during the initial months of the COVID-19 pandemic. An online survey | Lewis R, Blake C, Shimonovich M, Coia N, Duffy J, Kerr Y, Wilson J, Graham CA, Mitchell KR. Disrupted prevention: condom and contraception access and use among young adults during the initial months of the COVID-19 pandemic. An online survey. BMJ Sexual & Reproductive Health. 2021 Oct 1;47(4):269-76. | 2021 | The United Kingdom (Scotland). | *“Illuminate young people’s experiences of accessing and  using condoms and contraception in the early months of the pandemic.”* | Mixed Methods | **Data Collection:**  Quantitative and qualitative survey.  **Data Analysis:**  Descriptive statistics, inductive qualitative analyses using the Framework Method. | Youth aged 16-24 living in Scotland (n=2005) |
| E Hine: access to contraception for indigenous Maori teenage mothers | Lawton B, Makowharemahihi C, Cram F, Robson B, Ngata T. E Hine: Access to contraception for indigenous Māori teenage mothers. Journal of Primary Health Care. 2016 Mar 31;8(1):52-9. | 2016 | New Zealand (Wellington and Hawkes Bay) | *“To identify barriers and facilitators to contraception for Māori teenagers who become mothers.”* | Qualitative | **Data Collection:**  Pre-birth cohort interviewed 4-5 interviews over 20 months. Post-birth cohort interviewed 2-3 times over 9 months.  **Data Analysis:**  Two-cycle coding approach: 1) focused on participants' experiences, knowledge, attitudes, or values related to contraception to ; 2) focussed on salient features, further explore the themes from coding cycle one and seeking feedback from the research team. | Māori teenagers aged 14-19 who become mothers (pre-birth cohort n=22, post-birth cohort n=22, total n=44). |
| Examining Parental Acceptance of Confidential Contraception Initiation in a Pediatric Emergency Department | Kerolle S, Zuckerbraun N, Reed J, Pollack A, Lieberman J, Ruppert K, Hoehn E. Examining parental acceptance of confidential contraception initiation in a pediatric emergency department. Pediatr Emerg Care. 2022 Aug 5;39:1-5. | 2022 | The United States of America (Western Pennsylvania, Eastern Ohio, Western New York, Western Maryland). | *“(1) assess parental acceptance of confidential provisions of contraception in the PED and (2) identify facilitators/barriers to parental acceptance of contraception in this setting.”* | Quantitative | **Data Collection:**  Survey  **Data Analysis:**  Descriptive statistics, Chi-Squared or Fisher’s exact tests, t-test or Wilcoxon sign rank test. | Parents/guardians of females aged 14 to 19 in a pediatric emergency department (n=102). |
| Experiences of pregnancy prevention among youth experiencing homelessness | Eapen DJ, Bergh R, Lucas S, Narendorf SC, Begun S, Santa Maria D. Experiences of pregnancy prevention among youth experiencing homelessness. Children and Youth Services Review. 2023 Oct 1;153:107115. | 2023 | The United States of America (South). | *“What are the pregnancy prevention experiences of YEH?” and “How do YEH describe their sexual and reproductive health practices?”* | Qualitative | **Data Collection:**  Focus group discussions based on social cognitive theory framework.  **Data Analysis:**  States Inductive thematic content analysis in the abstract and inductive thematic approach based on social cognitive theory in the data analysis section. | Youth experiencing homelessness aged 18-25 from a city (n=81) |
| Exploring young women's reasons for adopting intrauterine or oral emergency contraception in the United States: a qualitative study | Kaller S, Mays A, Freedman L, Harper CC, Biggs MA. Exploring young women’s reasons for adopting intrauterine or oral emergency contraception in the United States: a qualitative study. BMC women's health. 2020 Dec;20:1-9. | 2020 | The United States of America (California). | To explore the “*factors that influence patients’ choice between emergency contraception pills and the copper IUD as emergency contraception, including prior experiences with contraception and attitudes toward emergency contraception methods, in settings where both options are available at no cost.”* | Qualitative | **Data Collection:**  Interviews with open and closed ended questions.  **Data Analysis:**  Thematic analysis approached with an interpretive constructivist lens. | Women under 30 seeking emergence contraception at three youth clinics (n=17) |
| From request to dispensation: how adolescent and young adult females experience access to emergency contraception in pharmacies | Barrense-Dias Y, Stadelmann S, Suris JC, Akre C. From request to dispensation: how adolescent and young adult females experience access to emergency contraception in pharmacies. The European Journal of Contraception & Reproductive Health Care. 2022 Sep 3;27(5):403-8. | 2022 | Europe (Switzerland). | *“To explore experiences of adolescent and young adult (AYA) females who have gone to a pharmacy in order to obtain EC [emergency contraception].”* | Qualitative | **Data Collection:**  Semi-structured interviews.  **Data Analysis:**  Inductive thematic content analysis. | Females (aged 15-25) who have gone to the pharmacy for emergency contraception in the caton of Vaud (n=30). |
| How can Primary Care Physicians Best Support Contraceptive Decision Making? A Qualitative Study Exploring the Perspectives of Baltimore Latinas | Carvajal DN, Gioia D, Mudafort ER, Brown PB, Barnet B. How can primary care physicians best support contraceptive decision making? A qualitative study exploring the perspectives of Baltimore Latinas. Women's Health Issues. 2017 Mar 1;27(2):158-66. | 2017 | The United States (Maryland). | *“This study used the theory of planned behavior as a guide to help describe Latinas’ perspectives regarding specific factors that influence their contraceptive decision making and to describe their perspectives about the role of PCPs [primary care providers] in the decision making.”* | Qualitative | **Data Collection:**  Focus groups and interviews.  **Data Analysis:**  Coding involved memoing, line-by-line coding, two researchers discussed their themes and concepts to theory of planned behaviour for data organization, initial code development, and coding scheme. A codebook with categories and subcategories was iteratively developed.  Concepts outlined theory of planned behaviour help to develop the coding scheme. | Latina immigrant youth ages 15 to 24 years in Baltimore (n=16). |
| Minors' Experiences Accessing Confidential Contraception in Texas | Whitfield B, Vizcarra E, Dane'el A, Palomares L, D'Amore G, Maslowsky J, White K. Minors' Experiences Accessing Confidential Contraception in Texas. Journal of Adolescent Health. 2023 Apr 1;72(4):591-8. | 2023 | The United States of America (Texas). | *“Texas minors’ reasons for and experiences seeking confidential contraception.”* | Qualitative | **Data Collection:**  In-depth interviews  **Data Analysis:**  Thematic analysis. | Texas youth aged 15-17 who tried to access contraception without parental involvement in the last year (n=28). |
| Perspectives on family planning services among adolescents at a Boston community health center | Johnson KM, Dodge LE, Hacker MR, Ricciotti HA. Perspectives on family planning services among adolescents at a Boston community health center. Journal of Pediatric and Adolescent Gynecology. 2015 Apr 1;28(2):84-90. | 2015 | The United States of America (Massachusetts). | *“To investigate adolescent perspectives on family planning services at a community-health center, with the intent to inform health center programs aimed at stemming the adolescent pregnancy rate, quality improvement.”* | Mixed Methods | **Data Collection:**  Semi-structured interviews and a survey.  **Data Analysis:**  Inductive qualitative analyses, descriptive statistics. | Adolescent females aged 16-20 who used services at a health center in Boston (n=20). |
| Primary care physicians' concerns may affect adolescents' access to intrauterine contraception | Rubin SE, Campos G, Markens S. Primary care physicians’ concerns may affect adolescents’ access to intrauterine contraception. Journal of primary care & community health. 2013 Jul;4(3):216-9. | 2013 | The United States of America (New York). | *“Explored primary care physicians’ (PCPs) approaches to contraception counseling with adolescents, focusing on their views about who would be appropriate IUD candidates.”* | Qualitative | **Data Collection:**  In-depth interviews.  **Data Analysis:**  Thematic analysis grouped outcomes into behaviours of counseling and inserting. A conceptual model was developed. | Healthcare practitioners (family physicians, pediatricians, and obstetrician gynecologists) who care for female youth a minimum of 30% of the time at an outpatient clinic, practice at a 1 of 2 large medical centres, and in an area with high adolescent pregnancy rates (n=28). |
| Racial and Ethnic Discrimination, Medical Mistrust, and Satisfaction with Birth Control Services among Young Adult Latinas | Oakley LP, Harvey SM, López-Cevallos DF. Racial and ethnic discrimination, medical mistrust, and satisfaction with birth control services among young adult Latinas. Women's Health Issues. 2018 Jul 1;28(4):313-20. | 2018 | The United States of America (Oregon). | *“Provide a better understanding of the social and structural factors that contribute to satisfaction with contraceptive services among Latinas living in rural areas.”* | Quantitative | **Data Collection:**  Computer assisted interview.  **Data Analysis:**  Descriptive statistics, bivariate associations (correlation), three-  step hierarchical, multivariable logistic regression models. | Latina women aged 18-25 in four rural counties who have ever seen a healthcare practitioner for contraception. |
| Seeking the female (internal) condom in retail pharmacies: Experiences of adolescent mystery callers | Hsu R, Tavrow P, Uysal J, Alterman AE. Seeking the female (internal) condom in retail pharmacies: Experiences of adolescent mystery callers. Contraception. 2020 Feb 1;101(2):117-21. | 2020 | The United States of America (  Arizona, California, New Mexico and Utah). | *“To describe barriers that adolescent mystery callers encountered when trying to access female condoms in U.S. pharmacies.”* | Mixed-Methods | **Data Collection:**  Mystery callers asking about pregnancy prevention and female condoms.  **Data Analysis:**  Responses were coded as 1) helpful, 2) no substantive comment, and 3) unhelpful. Descriptive statistics, differences were calculated through Chi-Square statistic. | Mystery callers posed as *16-year-olds seeking to avoid a pregnancy and learn about female condoms”*  Called pharmacies and spoke to pharmacists in four states (n=1475). |
| The Perfect Storm: Perceptions of Influencing Adults Regarding Latino Teen Pregnancy in Rural Communities | Barral RL, Brindis CD, Hornberger L, Trent M, Sherman AK, Ramirez M, Finocchario-Kessler S, Ramaswamy M. The Perfect Storm: Perceptions of Influencing Adults Regarding Latino Teen Pregnancy in Rural Communities. Maternal and child health journal. 2023 Apr;27(4):621-31. | 2023 | The United States of America (Kansas). | *“To describe community stakeholders’ knowledge, beliefs, and attitudes about Adolescent and Young Adults AYA’s SRH [sexual and reproductive health] needs in three rural Latino communities in Kansas.”* | Quantitative | **Data Collection:**  Written survey incorporating Theory of Planned Behaviour  **Data Analysis:**  Descriptive statistics, Chi-Square and Fisher’s exact  Tests. | Stakeholders to Adolescent and Young Adults sexual and reproductive health in the three rural Latino communities (n=55). |
| Understanding Barriers to Contraception Screening and Referral in Female Adolescents and Young Adults with Cancer | Lindsay SF, Woodhams EJ, White KO, Drainoni ML, Johnson NL, Yinusa-Nyahkoon L. Understanding barriers to contraception screening and referral in female adolescents and young adults with cancer. Journal of Adolescent and Young Adult Oncology. 2020 Feb 1;9(1):63-71. | 2020 | The United States of America (Northeast). | *“To understand oncology clinicians’ current practices and perceptions of barriers to screening and referring young women for adequate contraception during cancer treatment.”* | Qualitative | **Data Collection:**  Semi-structured interviews based on the Promoting Action on Research Implementation in Health Services (PARiHS) framework.  **Data Analysis:**  Directed content analysis and the constant comparative method. | Oncology clinicians from an urban Northeast medical centre (n=19). |
| Understanding Commercially Sexually Exploited Youths' Facilitators and Barriers toward Contraceptive Use: I Didn't Really Have a Choice | Kelly MA, Bath EP, Godoy SM, Abrams LS, Barnert ES. Understanding commercially sexually exploited youths' facilitators and barriers toward contraceptive use: I didn't really have a choice. Journal of pediatric and adolescent gynecology. 2019 Jun 1;32(3):316-24. | 2019 | The United States of America. | *“To understand facilitators and barriers related to their use of condoms and hormonal contraception for commercially sexually exploited youth.”* | Qualitative | **Data Collection:**  Semi-structured interviews.  **Data Analysis:**  Thematic analysis. | Commercially sexually exploited female youth (traded sex for something valuable) older than 12, younger than 23 (n=21). |
| Understanding the low uptake of long-acting reversible contraception by young women in Australia: A qualitative study | Garrett CC, Keogh LA, Kavanagh A, Tomnay J, Hocking JS. Understanding the low uptake of long-acting reversible contraception by young women in Australia: a qualitative study. BMC women's health. 2015 Dec;15(1):1-0. | 2015 | Australia (Victoria). | *“To investigate the barriers to young women’s use of LARC in Australia and to identify possible approaches for increasing LARC knowledge and access.”* | Qualitative | **Data Collection:**  Interviews (healthcare practitioners), focus groups (young women).  **Data Analysis:**  Thematic analysis. | Healthcare practitioners interested in young women’s reproductive health (n=15). Sexually active women aged 16-25 from metropolitan and regional areas of Victoria (n=27) |
| Unmet demand for short-acting hormonal and long-acting reversible contraception among community college students in Texas | Hopkins K, Hubert C, Coleman-Minahan K, Stevenson AJ, White K, Grossman D, Potter JE. Unmet demand for short-acting hormonal and long-acting reversible contraception among community college students in Texas. Journal of American College Health. 2018 Jul 4;66(5):360-8. | 2018 | The United States of America (Texas). | *“To identify preferences for and use of short-acting hormonal or long-acting reversible contraception among community college students in Texas.”* | Quantitative | **Data Collection:**  Survey  **Data Analysis:**  Descriptive statistics, Chi-Square test, and multivariable logistic regression. | Female community college students, ages 18 to 24, at risk of pregnancy, in Texas  (n=966). |
| Women's perceptions of pharmacist-prescribed hormonal contraception | Meredith AH, Olenik NL, Farris KB, Hudmon KS. Women's perceptions of pharmacist‐prescribed hormonal contraception. Journal of the American College of Clinical Pharmacy. 2020 Mar;3(2):425-32. | 2020 | The United States of America (Indiana). | *“Identify how women at risk for unintended pregnancy in central Indiana perceive pharmacist prescribing of contraception.”* | Qualitative | **Data Collection:**  Semi-structured interviews.  **Data Analysis:**  Iterative coding. Two researchers independently coded all transcripts and discussed till consensus. | Females, aged 18 to 29-years-old (n=14). |
| Youth Perspectives on Pharmacists' Provision of Birth Control: Findings From a Focus Group Study | Zuniga C, Wollum A, Katcher T, Grindlay K. Youth perspectives on pharmacists’ provision of birth control: findings from a focus group study. Journal of Adolescent Health. 2019 Oct 1;65(4):514-9. | 2019 | The United States of America (Washington DC) | *“This study explores the needs and concerns of teens and young women residing in Washington, D.C. to inform implementation of this service.”* | Qualitative | **Data Collection:**  Focus groups.  **Data Analysis:**  Inductive and deductive thematic analysis. | Females aged 14-24 in Washington DC (n=16). |
