# Supplementary material for: Barriers to contraception access and use among youth: A scoping review in high‐income countries
Source: Int J Gynaecol Obstet. 2025 Nov 14;173(1):74–86. doi: 10.1002/ijgo.70637 (PMC12988402; doi:10.1002/ijgo.70637)
Supplement: Supplementary file 4 — Table S2A. Mixed Methods Appraisal Tool Version 2018 Article Assessments for Qualitative and Quantitative Non‐Randomized Articles in this Scoping Review on Youth Contraception Barriers in High Income Countries (n = 41). Consensus reached between two assessors. Only Relevant Study Designs Pertaining to this Review are Included. Table S2B. Mixed Methods Appraisal Tool Version 2018 Article Assessments of Quantitative Descriptive and Mixed‐Methods Articles in this Scoping Review on Youth Contraception Barriers in High Income Countries (n = 41). Consensus reached between two assessors. Only Relevant Study Designs Pertaining to this Review are Included. [file IJGO-173-74-s001.docx]

| **Supplemental Table 2A (Table S2A).** Mixed Methods Appraisal Tool Version 2018 Article Assessments for Qualitative and Quantitative Non-Randomized Articles in this Scoping Review on Youth Contraception Barriers in High Income Countries (n=41). Consensus reached between two assessors. Only Relevant Study Designs Pertaining to this Review are Included. | | | | | | | | | | | | | |
| --- | --- | --- | --- | --- | --- | --- | --- | --- | --- | --- | --- | --- | --- |
|  | **All Articles** | | **1. Qualitative Articles (n= 20)** | | | | | **3. Quantitative Non-Randomized (n=2)** | | | | |  |
| **Article Title** | **S1. Are there clear research questions?** | **S2. Do the collected data allow to address the research questions?** | **1.1. Is the qualitative approach appropriate to answer the research question?** | **1.2. Are the qualitative data collection methods adequate to address the research question?** | **1.3. Are the findings adequately derived from the data?** | **1.4. Is the interpretation of results sufficiently substantiated by data?** | **1.5. Is there coherence between qualitative data sources, collection, analysis and interpretation?** | **3.1. Are the participants representative of the target population?** | **3.2. Are measurements appropriate regarding both the outcome and intervention (or exposure)?** | **3.3. Are there complete outcome data?** | **3.4. Are the confounders accounted for in the design and analysis?** | **3.5. During the study period, is the intervention administered (or exposure occurred) as intended?** | **Overall Article Quality**  **(%)** |
| "It seems kinda like a different language to us": Homeless youths' attitudes and experiences pertaining to condoms and contraceptives | **YES**  The research question of the study is clear and consistent. | **YES**  Data collection approaches align well with the research question to elaborate on homeless youth’s experiences and attitudes. | **YES**  A qualitative approach is appropriate to focus on youth’s experiences and attitudes. | **YES**  Purposeful sampling is adequate, and youth were consented in a safe an appropriate manner. Semi structured interviews align nicely. | **YES**  The qualitative analysis plan is appropriate. | **YES**  The findings are organized well, and the quotes justify the themes. | **YES**  Clear coherence throughout this article. |  |  |  |  |  | 100% |
| "The thing in my arm": Providing contraceptive services for adolescents in primary care | **YES**  Clear research questions. | **YES**  The data collected is appropriate. | **YES**  A qualitative approach is appropriate. | **YES**  The data collected is appropriate. | **YES**  A grounded theory approach is appropriate. | **YES**  Lots of quote that describe the themes. | **YES**  Good coherence throughout. |  |  |  |  |  | 100% |
| A Pediatric Emergency Department Intervention to Increase Contraception Initiation Among Adolescents | **YES**  Clear research aim. | **YES**  The data collected is appropriate. |  |  |  |  |  | **YES**  Appropriate convenience sample. | **YES**  Clear measures. | **NO**  Missing outcome data. | **NO**  Confounders are not mentioned. | **CAN’T TELL**  Unclear based on study descriptions. | 20% |
| A Qualitative Analysis of Long-Acting Reversible Contraception | **YES**  Clear research question. | **YES**  The data collected addresses the research aim. | **YES**  A qualitative approach is appropriate. | **YES**  The data collected addresses the research question. | **YES**  The qualitative analysis approach is adequate. | **YES**  The data provided aligns with the presented themes. | **YES**  Well aligned study. |  |  |  |  |  | 100% |
| A Qualitative Assessment to Understand the Barriers and Enablers Affecting Contraceptive Use Among Adolescent Male Emergency Department Patients | **YES**  The research objectives are clear. | **YES**  The data collected is appropriate. | **YES**  A qualitative approach is appropriate. | **YES**  Data collected are sufficient. | **YES**  Thematic analysis is a good approach. | **YES**  The quotes justify the themes sufficiently. | **YES**  Clear alignment throughout the article. |  |  |  |  |  | 100% |
| A Qualitative Study Exploring Contraceptive Practices and Barriers to Long-Acting Reversible Contraceptive Use in a Sample of Adolescents Living in the Southern United States | **YES**  There is a clear study objective. | **YES**  The data collected is appropriate. | **YES**  A qualitative approach is appropriate. | **CAN’T TELL**  Hard to gather data in 15 minutes. Non-compensation assumptions were inappropriate. | **CAN’T TELL**  There is a discrepancy between the type of analysis done. In the abstract it says qualitative content analysis, but in the methods section, it says transcripts were coded using grounded theory. They also do not describe their qualitative process in much detail. | **YES**  The limited quotes provide justification | **YES**  Coherence throughout. |  |  |  |  |  | 60% |
| A State-Level Examination of School Nurses' Perceptions of Condom Availability Accompanied by Sex Education | **YES**  Appropriate study design. | **YES**  Appropriate collected data. | **YES**  A qualitative approach is appropriate. | **NO**  Minimally collected qualitative questions | **CAN’T TELL**  The analysis plan needed to be more specific. | **YES**  Quotes are provided to justify the themes. | **YES**  There is coherence throughout the study. |  |  |  |  |  | 60% |
| Assessing perspectives on an intervention connecting adolescents in outpatient psychiatry care to contraceptive counseling in the United States | **YES**  Appropriate study design. | **YES**  Appropriate collected data. | **YES**  A qualitative approach is appropriate. | **YES**  The data collected is appropriate. | **YES**  The qualitative analysis approach is adequate. | **YES**  Quotes are provided to justify the themes. | **YES**  There is coherence throughout the study. |  |  |  |  |  | 100% |
| Availability of Confidential Services for Teens Declined After the 2011–2013 Changes to Publicly Funded Family Planning Programs in Texas | **YES** There is a clear objective. | **YES**  The data collected addresses the objective. | **YES**  A qualitative approach is appropriate. | **YES**  The data collected is appropriate. | **YES**  The qualitative analysis approach is appropriate. | **YES**  The quotes align well with the themes. | **YES**  There is adequate coherence throughout. |  |  |  |  |  | 100% |
| Barriers to and enablers of contraceptive use among adolescent females and their interest in an emergency department-based intervention | **YES**  Clear research objectives provided. | **YES**  The data collected is appropriate. | **YES**  A qualitative approach is appropriate. | **YES**  Data collection is a good approach to address the research objectives. | **CAN’T TELL**  More clarification into their analysis plan is needed. | **YES** The quotes justified the themes sufficiently. | **YES**  Coherence throughout. |  |  |  |  |  | 80% |
| Barriers to Long-Acting Reversible Contraceptive Uptake Among Homeless Young Women | **YES**  Clear research question. | **YES**  The data collected is appropriate. | **YES**  A qualitative approach is appropriate. | **CAN’T TELL**  While the interview guide was not provided, they outline questions asked which are relevant to the research objective. It was unclear how the data was recorded. | **CAN’T TELL**  It is unclear if a deductive coding approach is appropriate for the research objective to explore youth’s experiences. No rationale for why a deductive approach is provided. | **YES**  There are a lot of good quotations that highlight the data themes. | **YES**  Clear links throughout the study. |  |  |  |  |  | 60% |
| Can youth get the contraception they want? Results of a pilot study in the province of Quebec | **YES**  Clear research question. | **YES**  The data collected is appropriate. | **NO**  The qualitative approach is minimal and unclear and therefore is not adequate to address the research question. | **NO**  Free-text survey questions are sufficient for understanding "Quebec youth’s experience of obtaining contraception." | **CAN’T TELL**  It is unclear how they conducted qualitative analysis on the free-response questions. | **CAN’T TELL**  The presentation of qualitative quotes to inform study themes is unclear. Additionally, the presentation of qualitative data is challenging to follow. | **CAN’T TELL**  Hard to answer because the analysis plan is not clear and it is challenging to understand how the qualitative data informed theme development. |  |  |  |  |  | 0% |
| Concerns About the Cost of Contraception Among Young Women Attending Community College | **YES**  Clear research aim. | **YES**  The data collected is appropriate. |  |  |  |  |  | **YES**  Reached out to community colleges which reflect the target population. | **YES**  Appropriate measurements. Clear outcomes, exposure, and control variables. | **CAN’T TELL**  Could have been more explicit. There were 190 participants concerned with contraception costs, but it is unknown if this was everyone. | **YES**  Accounted for confounders. | **CAN’T TELL**  Not explicit, but assumed it went okay. | 60% |
| Disrupted prevention: condom and contraception access and use among young adults during the initial months of the COVID-19 pandemic. An online survey | **YES**  Clear research objective. | **YES**  Appropriate data collection approach. | **YES**  A qualitative approach is appropriate. | **NO**  It feels insufficient to conceptualize "people’s experiences of accessing and using condoms and contraception in the early months of the pandemic" with just one open response question. | **YES**  Clear qualitative analysis approach that is appropriate for the study. | **YES**  Findings are summarized well and good quotes throughout. | **YES**  Clear coherence and process. |  |  |  |  |  | 100% |
| E Hine: access to contraception for indigenous Maori teenage mothers | **YES**  Clear research aim. | **YES**  The data collected is appropriate. | **YES**  A qualitative approach is appropriate. | **YES**  Large qualitative study. | **YES**  Good description of analysis plan. | **YES**  The quotes justify the themes sufficiently. | **YES**  Coherence throughout. |  |  |  |  |  | 100% |
| Examining Parental Acceptance of Confidential Contraception Initiation in a Pediatric Emergency Department | **YES**  Clear research aim/question. | **YES**  The data collected reflects the research aim. | **YES**  A qualitative approach is appropriate. | **YES**  The data collected is adequate. | **YES**  Good description and design of their analytic approach. | **YES**  Interpretations summarize data well. | **YES**  Clear coherence throughout. |  |  |  |  |  | 100% |
| Experiences of pregnancy prevention among youth experiencing homelessness | **YES**  Clear research questions. | **YES**  The data collected are appropriate. | **YES**  A qualitative approach is an appropriate. | **YES**  The data collected align with the research objectives. | **YES**  Good analytical approach. | **YES**  The quotes provided justify the themes. | **YES**  Good alignment throughout. |  |  |  |  |  | 100% |
| Exploring young women's reasons for adopting intrauterine or oral emergency contraception in the United States: a qualitative study | **YES**  Clear research aim/question. | **YES**  The data collected addresses the research question. | **YES**  A qualitative approach is appropriate. | **YES**  The data collected is appropriate. | **YES**  The analytical approach is appropriate. | **YES**  Fair interpretations. | **YES**  Well organized study. |  |  |  |  |  | 100% |
| From request to dispensation: how adolescent and young adult females experience access to emergency contraception in pharmacies | **YES**  The aim is clear and consistent throughout the paper. | **YES**  Everything is tailored to addressing the research question/aim. | **YES**  An exploratory qualitative approach is appropriate. | **YES**  Study recruitment, and data collection approaches align well. | **YES**  A thematic content analysis with an inductive approach is appropriate. | **YES**  The quotes and descriptions justify the themes provided. | **YES**  The study is cohesive and aligned throughout. |  |  |  |  |  | 100% |
| How can Primary Care Physicians Best Support Contraceptive Decision Making? A Qualitative Study Exploring the Perspectives of Baltimore Latinas | **YES**  The objective is clear and consistent throughout the paper. | **YES**  The collected qualitative data aligns well with the research objective. | **YES**  A qualitative approach is appropriate to address the research question. | **YES**  The use of interviews and focus group is appropriate. Having a bilingual interviewer is helpful to ensure youth can speak freely and are not constrained by language. | **YES**  Their qualitative analysis approach was clear. However, they have minimal citations in the analysis section of their methods section to help further validate their approach. | **YES**  The quotes and descriptions encompass the presented themes. | **YES**  There is a clear story and cohesion throughout this article. |  |  |  |  |  | 100% |
| Minors' Experiences Accessing Confidential Contraception in Texas | **YES**  Clear research question. | **YES**  The data collected reflects the research question. | **YES**  A qualitative approach is appropriate. | **YES**  The data collected is appropriate. | **YES**  Their qualitative analysis plan is adequate. | **YES**  Lots of quotations to justify the presented themes. | **YES**  The study aligns nicely. |  |  |  |  |  | 100% |
| Perspectives on family planning services among adolescents at a Boston community health center | **YES**  Clear study aim. | **YES**  Data collected align with the study aim. | **YES**  Appropriate qualitative approach. | **YES**  The data collected is appropriate. | **YES**  Their qualitative analysis plan is adequate. | **YES**  Each theme is justified by a quote. | **YES**  Clear coherence throughout. |  |  |  |  |  | 100% |
| Primary care physicians' concerns may affect adolescents' access to intrauterine contraception | **YES**  Clear research objective/question. | **YES**  The data collected addresses the research question. | **CAN’T TELL**  They do not describe the qualitative approach(es) adequately. | **CAN’T TELL**  Did not describe the general time of each phone call. | **YES**  Published protocol. | **CAN’T TELL**  Not adequately described. | **YES**  Clear coherence throughout. |  |  |  |  |  | 40% |
| Seeking the female (internal) condom in retail pharmacies: Experiences of adolescent mystery callers | **YES**  Clear research objective/question. | **YES**  The data collected addresses the research question. | **YES**  A qualitative approach is appropriate. | **YES**  The data collection approach is adequate. | **CAN’T TELL**  Unclear how the qualitative coding was done. Unclear for how the three categories of an interaction were developed and how data was treated if they did not fall under these categories. | **YES**  The quotes justify the themes. | **YES**  Clear coherence throughout. |  |  |  |  |  | 80% |
| Understanding Barriers to Contraception Screening and Referral in Female Adolescents and Young Adults with Cancer | **YES**  Clear research question/aim. | **YES**  The data collection is appropriate. | **YES**  A qualitative approach is appropriate. | **YES**  The data collection approach is adequate. | **YES**  Good approach to qualitative analysis. | **YES**  The quotes justify the themes. | **YES**  Clear process and alignment throughout. |  |  |  |  |  | 100% |
| Understanding Commercially Sexually Exploited Youths' Facilitators and Barriers toward Contraceptive Use: I Didn't Really Have a Choice | **YES**  Clear research aim. | **YES**  The data collected is a good choice. | **CAN’T TELL**  Their qualitative approach was a community-partnered and "in-depth" qualitative study. I am not sure of what approach(es) they specifically used. | **YES**  The data collected is appropriate. | **YES**  Appreciate to use a thematic analysis approach. Clear qualitative analysis plan. | **YES**  Interpretations are rooted in presented study data. | **YES**  Overall good organization and coherence. |  |  |  |  |  | 80% |
| Understanding the low uptake of long-acting reversible contraception by young women in Australia: A qualitative study | **YES**  There is a clear study aim. | **YES**  The data collected is appropriate. | **YES**  A qualitative approach is appropriate. | **YES**  A good approach, however unclear why focus groups and interviews were done. | **YES**  The qualitative approach is appropriate. | **YES**  The quotes justify the themes. | **YES**  Well organized and coherent. |  |  |  |  |  | 100% |
| Women's perceptions of pharmacist-prescribed hormonal contraception | **YES**  Clear research aim. | **YES**  Collected data address research objective. | **YES**  A qualitative approach is appropriate. | **YES**  The data collected is appropriate. | **CAN’T TELL**  Qualitative analysis plan is brief and does not provide much detail beyond an iterative approach. | **YES**  The quotes justify the themes. | **YES**  Well organized and coherent. |  |  |  |  |  | 80% |
| Youth Perspectives on Pharmacists' Provision of Birth Control: Findings from a Focus Group Study | **YES**  Clear research aim. | **YES**  Collected data address research objective. | **YES**  A qualitative approach is appropriate. | **YES**  The use of focus groups is appropriate. | **YES**  Appropriate qualitative analysis plan. | **YES**  The quotes justify the themes. | **YES**  There is coherence throughout the study. |  |  |  |  |  | 100% |

| **Supplemental Table 2B (Table S2B).** Mixed Methods Appraisal Tool Version 2018 Article Assessments of Quantitative Descriptive and Mixed-Methods Articles in this Scoping Review on Youth Contraception Barriers in High Income Countries (n=41). Consensus reached between two assessors. Only Relevant Study Designs Pertaining to this Review are Included. | | | | | | | | | | | | | | |
| --- | --- | --- | --- | --- | --- | --- | --- | --- | --- | --- | --- | --- | --- | --- |
|  | **All Articles** | | **4. Quantitative Descriptive (n=13)** | | | | | **5. Mixed-Methods (n=6)** | | | | |  |  |
| **Article Title** | **S1. Are there clear research questions?** | **S2. Do the collected data allow to address the research questions?** | **4.1. Is the sampling strategy relevant to address the research question?** | **4.2. Is the sample representative of the target population?** | **4.3. Are the measurements appropriate?** | **4.4. Is the risk of nonresponse bias low?** | **4.5. Is the statistical analysis appropriate to answer the research question?** | **5.1. Is there an adequate rationale for using a mixed methods design to address the research question?** | **5.2. Are the different components of the study effectively integrated to answer the research question?** | **5.3. Are the outputs of the integration of qualitative and quantitative components adequately interpreted?** | **5.4. Are divergences and inconsistencies between quantitative and qualitative results adequately addressed?** | **5.5. Do the different components of the study adhere to the quality criteria of each tradition of the methods involved?** | **Overall Article Quality**  **(%)** | **Overall Mixed-Methods Article Quality**  **(%)** |
| "I don't know enough to feel comfortable using them:" Women's knowledge of and perceived barriers to long-acting reversible contraceptives on a college campus | **YES**  Clear question. | **YES**  Collected data reflect research question. | **YES**  Appropriate sampling strategy. | **YES**  Survey of the target population of college women. | **YES**  Clear and appropriate measures. | **NO**  Low response rate. | **YES**  Appropriate statistical analyses. |  |  |  |  |  | 80% |  |
| A mystery shopper study identifying practice-level barriers to adolescent IUD access in western Pennsylvania | **YES**  Clear question. | **YES**  Collected data reflect research question. | **YES**  Appropriate sampling techniques. | **YES**  Appropriate sample. | **YES**  Clear measures. | **CAN’T TELL**  It's unclear how they obtained the 56 practices and if that's a complete list of practices. | **YES**  Appropriate statistical analyses. |  |  |  |  |  | 80% |  |
| A State-Level Examination of School Nurses' Perceptions of Condom Availability Accompanied by Sex Education | Mixed methods study (see Supplemental Table 3A). | Mixed methods study (see Supplemental Table 3A). | **YES**  Appropriate sampling strategy. | **YES**  Appropriate approach. | **YES**  Measurements are justified and appropriate for answering the research question. | **NO**  Poor response rate, risk of nonresponse bias high. | **YES**  Appropriate approach to analyses. | **NO**  No rationale was presented. | **NO**  No description into the approaches for integration. | **NO**  No integration is mentioned in the report. | **YES**  No divergences were apparent. | **NO**  Areas of improvement for both quantitative and qualitative approaches. |  | 20% |
| Access to Emergency Contraception After Removal of Age Restrictions | **YES**  Clear research question. | **YES**  The data collected is appropriate. | **YES**  Appropriate sampling strategy. | **YES**  Representative sample. | **YES**  Measurements are appropriate. | **YES**  Low response bias. | **YES**  Appropriate approach to analyses. |  |  |  |  |  | 100% |  |
| Access to Reproductive Health Care in Juvenile Justice Facilities | **YES**  Clear research aim. | **YES**  The data collected is appropriate. | **YES**  Appropriate sampling strategy. | **CAN’T TELL**  50 states were reached out to. But it is unclear why youth were not invited to participate, for example if they would be prohibited from participating. | **YES**  Measurements are appropriate. | **NO**  Low response rate (20/50). | **YES**  The quantitative analysis plan is appropriate. |  |  |  |  |  | 60% |  |
| Adolescents' and Young Adults' Reports of Barriers to Confidential Health Care and Receipt of Contraceptive Services | **YES**  Clear study purpose. | **YES**  The data collected is appropriate. | **CAN’T TELL**  They use national survey data, but information of how this survey was originally administrated was not clear. | **YES**  Sample was representative of target population; every pharmacy was contacted. | **YES**  Clear and appropriate measures. | **CAN’T TELL**  Survey response rates not mentioned. | **YES**  The quantitative analysis plan is appropriate. |  |  |  |  |  | 60% |  |
| Adolescents' perceived barriers to accessing sexual and reproductive health services in California: a cross-sectional survey | **YES**  Clear research question. | **YES**  The data collected is appropriate. | **YES**  The sampling strategy is clear and appropriate. | **YES**  Large representative sample. | **YES**  Good and aligned study measures. | **YES**  High response rate of 77.8% | **YES**  The quantitative analysis plan is appropriate. |  |  |  |  |  | 100% |  |
| Availability and Accessibility of Emergency Contraception to Adolescent Callers in Pharmacies in Four Southwestern States | **YES**  Clear research purpose. | **YES**  Appropriate data collection. | **YES**  Appropriate sampling strategy. | **YES**  Representative sample. | **YES**  Appropriate measurements. | **YES**  Low risk for nonresponse bias. | **YES**  Appropriate quantitative analyses. |  |  |  |  |  | 100% |  |
| Barriers to adolescent contraception use and adherence | **YES**  Clear research aim. | **YES**  The data collected is appropriate. | **YES**  The sampling strategy was appropriate. | **NO**  Only having one hospital centre is likely not representative of the target population of an ethnically diverse urban population. | **YES**  Clear survey and measures. | **NO**  A high nonresponse rate. | **CAN’T TELL**  Appropriate emphasis on descriptive statistics, but it is unclear why so many inferential approaches were chosen and which tests were used to determine statistical significance for the findings presented in the results. |  |  |  |  |  | 40% |  |
| Barriers to Long-Acting Reversible Contraceptive Uptake Among Homeless Young Women | Mixed methods study (see Supplemental Table 3A). | Mixed methods study (see Supplemental Table 3A). | **YES**  Appropriate sampling approach. | **NO**  The study only takes place in Pittsburgh and does not represent the target population. | **YES**  The measures were appropriate. | **CAN’T TELL**  The risk of nonresponse is high as only flyers were handed out at certain areas. It is unclear how many youth were approached and how many responded. | **YES**  Appropriate quantitative analyses. | **NO**  Rationale for using mixed methods is not clear. | **NO**  Integration is not mentioned in the study. | **NO**  Outputs are not integrated. | **YES**  No divergences mentioned. | **NO**  The quality of the quantitative and qualitative pieces were not met. |  | 20% |
| Can youth get the contraception they want? Results of a pilot study in the province of Quebec | Mixed methods study (see Supplemental Table 3A). | Mixed methods study (see Supplemental Table 3A). | **NO**  Convenience sample is not the best approach as it is has a higher risk of bias. Posting study flyers in many public places does not feel like a convenience sampling approach. | **NO**  While the sampling is biased, the sample was small at 105, and it is unclear where in Quebec the survey reaches. | **CAN’T TELL**  While they state it is a survey, it is challenging to see what the survey questions are, but glimpses are provided into what participants were asked. | **CAN’T TELL**  Survey nonresponse was not discussed. For example, it is unclear what the target foot traffic is in areas where the study was posted and how many youth were expected to be eligible. | **YES**  Descriptive statistics and Chi-squared were appropriate. Geomapping was interesting to see where participants were from. | **NO**  There is no justification for using a mixed methods design to address the research question. | **NO**  Data integration is not discussed | **CAN’T TELL**  The study does not really touch on this. The qualitative findings are presented in a table. | **YES**  No divergence is mentioned. | **NO**  There are areas of improvement with respect to both the quantitative and qualitative branches of this study. |  | 20% |
| Delayed Visits for Contraception Due to Concerns Regarding Pelvic Examination Among Women with History of Intimate Partner Violence | **YES**  Clear research aim. | **YES**  The data collected is appropriate. | **YES**  Sampling strategy is appropriate. | **YES**  Data came across 40 reproductive health centres (n=1490), representative of the target population. | **YES**  Clear measures that are justified. | **CAN’T TELL**  It is unclear if there was nonresponse during initial data collection. | **YES**  Appropriate analytical plan. |  |  |  |  |  | 60% |  |
| Disrupted prevention: condom and contraception access and use among young adults during the initial months of the COVID-19 pandemic. An online survey | Mixed methods study (see Supplemental Table 3A). | Mixed methods study (see Supplemental Table 3A). | **CAN’T TELL**  Tried to find information regarding how the convenience sample was developed but could not easily find. | **NO**  The sample is large, but the study stated the goal to illuminate young people’s experiences. Therefore, the sample is not representative. | **YES**  Appropriate measures. | **CAN’T TELL**  Unclear. | **YES**  The quantitative analysis plan is not clear but from the results it is discerned to be descriptive statistics which is appropriate. | **YES**  Adequate rationale for mixed methods. | **CAN’T TELL**  Integration is not clearly mentioned or depicted in this report. | **CAN’T TELL**  This is not clearly depicted. | **YES**  No divergences reported. | **NO**  There are areas of improvement with the quantitative and qualitative components as well as integration. |  | 20% |
| Examining Parental Acceptance of Confidential Contraception Initiation in a Pediatric Emergency Department | **YES**  Clear research question. | **YES**  Data collection is appropriate. | **YES**  Sampling strategy is appropriate. | **YES**  Sample population is adequate for study. | **YES**  Clear and appropriate measures. | **YES**  Risk of nonresponse is low. | **YES**  Appropriate quantitative analyses for the sample. |  |  |  |  |  | 100% |  |
| Perspectives on family planning services among adolescents at a Boston community health center | Mixed methods study (see Supplemental Table 3A). | Mixed methods study (see Supplemental Table 3A). | **YES**  Sampling strategy is relevant. | **YES**  Sample is consistent with target population. | **CAN’T TELL**  Not much information provided. | **CAN’T TELL**  Nonresponse bias is not discussed. | **YES**  Descriptive statistics are appropriate given the small study size. | **YES**  Rationale provided. | **NO**  Analyses were not integrated, however the quantitative and qualitative findings are presented together. | **YES**  Quantitative and qualitative finding are blended throughout the results. | **YES**  No divergences reported. | **NO**  The quantitative component as not as strong as the qualitative work. |  | 60% |
| Racial and Ethnic Discrimination, Medical Mistrust, and Satisfaction with Birth Control Services among Young Adult Latinas | **YES**  Clear research aim. | **YES**  Appropriate data collection methods. | **YES**  Appropriate sampling approaches. | **YES**  Representative sample of Latina women in rural Oregon. | **YES**  Measurements are appropriate and justified. | **CAN’T TELL**  Unclear what the eligibility pool size overall was from those screened | **YES**  Good analytical approach. |  |  |  |  |  | 80% |  |
| Seeking the female (internal) condom in retail pharmacies: Experiences of adolescent mystery callers | Mixed methods study (see Supplemental Table 3A). | Mixed methods study (see Supplemental Table 3A). | **YES**  Pharmacy recruitment was clear and good. However, it is unclear how the youth who made the calls were recruited. | **YES**  Sample is representative as there were a large number of pharmacies included across several states. | **YES**  Appropriate and clear measures. | **YES**  High response rate of 86%. | **YES**  Good analytical approach. | **NO**  There is no real rationale for a mixed methods design. | **NO**  The approach to data integration was not discussed. | **NO** Outputs are not integrated. | **YES**  No divergences reported. | **NO**  There are areas of improvement for both the quantitative and qualitative components. |  | 20% |
| The Perfect Storm: Perceptions of Influencing Adults Regarding Latino Teen Pregnancy in Rural Communities | **YES**  The study aim is clear. | **YES**  The collected data is appropriate to address the research question. | **YES**  Appropriate sampling approach. | **YES**  They described target population adequately and justified their sample. The sample was a respective size. | **YES**  The measures are appropriate | **YES**  Low risk of non-response bias. | **CAN’T TELL**  Descriptive statistics appropriate. It is unclear why they did a test for statistical significance (using Chi Squared and Fisher’s Exact Test) with a small sample size. |  |  |  |  |  | 80% |  |
| Unmet demand for short-acting hormonal and long-acting reversible contraception among community college students in Texas | **YES**  Clear study objective. | **YES**  Appropriate data collected. | **YES**  Sampling strategy is relevant. | **NO**  Not all students had the chance to participate as participants were randomly selected. | **YES**  Study measures are appropriate. | **NO**  Low response rate | **YES**  Quantitative analysis plan is adequate. |  |  |  |  |  | 60% |  |
