# Supplementary material for: Barriers to contraception access and use among youth: A scoping review in high‐income countries
Source: Int J Gynaecol Obstet. 2025 Nov 14;173(1):74–86. doi: 10.1002/ijgo.70637 (PMC12988402; doi:10.1002/ijgo.70637)
Supplement: Supplementary file 5 — Table S3. List of Included Demographics in this Scoping Review on Youth Contraception Barriers in High Income Countries (n = 41). [file IJGO-173-74-s004.docx]

| **Supplemental Table 3 (Table S3).** List of Included Demographics in this Scoping Review on Youth Contraception Barriers in High Income Countries (n=41) | | | | | | | | | | | | |
| --- | --- | --- | --- | --- | --- | --- | --- | --- | --- | --- | --- | --- |
| **Article Title** | **Age of Youth** | **Sex & Gender of Youth** | **Sexual Orientation of Youth** | **Race, Ethnicity, and Indigeneity of Youth** | **Socioeconomic Status of Youth/Community** | **Geography** | **Community Size** | **Youth Relationship Status** | **Youth Education Level** | **Youth Employment** | **Youth Religion** | **Other Demographics** |
| "I don't know enough to feel comfortable using them:" Women's knowledge of and perceived barriers to long-acting reversible contraceptives on a college campus | Yes | Did not specify, only said women. | Yes | Yes | Not discussed | Yes | Not clear. | Yes | Yes | Yes | Yes | live on campus, participation in sorority, health insurance status, mother characteristics. |
| "It seems kinda like a different language to us": Homeless youths' attitudes and experiences pertaining to condoms and contraceptives | Yes | Gender | Yes | Yes | Yes | Yes | Yes Urban. | No | No | No | No | Foster care history, transience, pregnancy status, and pregnancy attitudes. |
| "The thing in my arm": Providing contraceptive services for adolescents in primary care | N/A practice administrators | N/A practice administrators | N/A practice administrators | N/A practice administrators | N/A practice administrators | Yes | Yes. Mix of urban and rural. | N/A practice administrators | N/A practice administrators | N/A practice administrators | N/A practice administrators |  |
| A mystery shopper study identifying practice-level barriers to adolescent IUD access in western Pennsylvania | Mystery Caller Posed 16-year-old females | Only stated female. | N/A mystery shopper study | N/A mystery shopper study | N/A mystery shopper study | Not explicit. | Not clear. | N/A mystery shopper study | N/A mystery shopper study | N/A mystery shopper study | N/A mystery shopper study | Minimal as it is brief research article about mystery shopper. |
| A Pediatric Emergency Department Intervention to Increase Contraception Initiation Among Adolescents | Yes | Only stated female. | Discusses sexual activity as opposed to sexual orientation. | Yes | No | Not explicit. | Yes Urban. | Yes | No | No | No | Insurance Status. |
| A Qualitative Analysis of Long-Acting Reversible Contraception | Yes | Only stated women. | Yes | Yes | Not discussed. | Not explicit. | Not clear. | Yes | Yes | No | No | Sexually active with another person. |
| A Qualitative Assessment to Understand the Barriers and Enablers Affecting Contraceptive Use Among Adolescent Male Emergency Department Patients | Yes | Only stated males. | Not discussed but needed to be sexually active with females. | Yes | Yes | Yes | Yes Urban. | Not explicit but discussed through sexual history | No | No | No | Insurance status, past use of medical care, and sexual history. |
| A Qualitative Study Exploring Contraceptive Practices and Barriers to Long-Acting Reversible Contraceptive Use in a Sample of Adolescents Living in the Southern United States | Yes | Only stated girls. | Not discussed. | Yes | Not discussed. | Yes | Yes Urban. | Not explicit, but need to be sexually active. | No | No | No | Insurance status. |
| A State-Level Examination of School Nurses' Perceptions of Condom Availability Accompanied by Sex Education | N/A nurses | N/A nurses | N/A nurses | N/A nurses | N/A nurses | Yes | Yes. Mix of urban and rural. | N/A nurses | Nurses in high schools. | N/A nurses | N/A nurses | Nurse participant demographics: age, education, sex education at school associated with. |
| Access to Emergency Contraception After Removal of Age Restrictions | Mystery callers posing as 17-year-old adolescents. | Only stated female. | N/A mystery shopper study. | N/A mystery shopper study. | Yes | Yes | Yes Urban. | N/A mystery shopper study. | N/A mystery shopper study. | N/A mystery shopper study. | N/A mystery shopper study. |  |
| Access to Reproductive Health Care in Juvenile Justice Facilities | State-level health care administrators in juvenile justice systems. | State-level health care administrators in juvenile justice systems, not discussed. | State-level health care administrators in juvenile justice systems, not discussed. | State-level health care administrators in juvenile justice systems, not discussed. | N/A State-level health care administrators in juvenile justice | Yes | Not clear. Across America. | N/A State-level health care administrators in juvenile justice | N/A State-level health care administrators in juvenile justice | N/A State-level health care administrators in juvenile justice | N/A State-level health care administrators in juvenile justice | Characteristics of Respondents Health care delivery arrangement, facilities represented, respondent role. |
| Adolescents' and Young Adults' Reports of Barriers to Confidential Health Care and Receipt of Contraceptive Services | Yes | Sex (male or female) | Not discussed | Yes | Yes | Not explicit. | Not clear. | Not explicit but states sexual experiences. | No | No | No | Current living arrangement, characteristics of mother, sexual health education, use of sexual health services in the past 12 months. |
| Adolescents' perceived barriers to accessing sexual and reproductive health services in California: a cross-sectional survey | Yes | Gender | Yes | Yes | Yes | Yes | Yes. Mix of urban and rural. | No | No | No | No | Living situation, ever had vaginal and/or anal sex. |
| Assessing perspectives on an intervention connecting adolescents in outpatient psychiatry care to contraceptive counseling in the United States | Not collected but 14 or older. | Assigned female at birth. | No | No | No | Yes | Yes Urban. | No | No | No | No |  |
| Availability and Accessibility of Emergency Contraception to Adolescent Callers in Pharmacies in Four Southwestern States | mystery callers posing as 16-year-old adolescents. | Only stated males and females. | Stated heterosexual. | Not mentioned, mystery caller study. | No | Yes | Yes. Mix of urban and rural. | No | No | No | No |  |
| Availability of Confidential Services for Teens Declined After the 2011–2013 Changes to Publicly Funded Family Planning Programs in Texas | N/A Program administrators. | N/A Program administrators. | N/A Program administrators. | N/A Program administrators. | N/A Program administrators. | Yes | Not clear. Stated across Texas. | N/A Program administrators. | N/A Program administrators. | N/A Program administrators. | N/A Program administrators. |  |
| Barriers to adolescent contraception use and adherence | Yes | Stated female. | No | Yes | No | Not explicit | Yes Urban. | Yes | Yes | Yes | No | Insurance, primary language, internet access, contraception specific questions |
| Barriers to and enablers of contraceptive use among adolescent females and their interest in an emergency department based intervention | Yes | Stated female | No | No, but the sample was largely Hispanic. | Yes | Not explicit | Yes Urban. | Yes | Yes | Yes | No | Lived in the United States, health insurance, access to healthcare, contraception questions, prior pregnancies |
| Barriers to Long-Acting Reversible Contraceptive Uptake Among Homeless Young Women | Yes | Stated female | Not mentioned but states sexual partners. | Yes | Yes | Yes | Yes Urban. | Yes | No | No | No | Currently pregnant, current pregnancy intention, current contraception use, experienced reproductive and/or coercion. |
| Can youth get the contraception they want? Results of a pilot study in the province of Quebec | Yes | Gender | No | No | No | Yes | Not clear. Did mention across the province. | No | Yes | Yes | No | Language preference, private insurance, contraception ever used, and source of payment. |
| Concerns About the Cost of Contraception Among Young Women Attending Community College | Yes | Self-identified females | Sexual identity | Yes | Yes | Yes | Not clear. Did mention college size, but not surrounding community. | No | Yes | No | No | Foreign born, has children, first generation college student, and type of insurance. |
| Delayed Visits for Contraception Due to Concerns Regarding Pelvic Examination Among Women with History of Intimate Partner Violence | Yes | Stated women | No | Yes | No | Yes | Not clear. | Yes | Yes | No | No | Insurance, and practice setting. |
| Disrupted prevention: condom and contraception access and use among young adults during the initial months of the COVID-19 pandemic. An online survey | Yes | Gender identity | Yes | Yes | Yes | Yes | Not clear. | No | No | No | No | Disability physical or mental health condition, and main living arrangement in the past year. |
| E Hine: access to contraception for indigenous Maori teenage mothers | Yes | Stated women. | No | Yes | No | Yes | Yes Urban. | No | No | No | No |  |
| Examining Parental Acceptance of Confidential Contraception Initiation in a Pediatric Emergency Department | Yes | Stated adolescent women. | N/A parents/guardian | N/A parents/guardian | N/A parents/guardian | Yes | Yes Urban. | N/A parents/guardian | N/A parents/guardian | N/A parents/guardian | N/A parents/guardian | Parental demographics: age, race, ethnicity, education, relationship to teen, questions about se and reproductive healthcare |
| Experiences of pregnancy prevention among youth experiencing homelessness | Yes | Gender | No | Yes | Yes | Yes | Yes Urban. | No | No | No | No |  |
| Exploring young women's reasons for adopting intrauterine or oral emergency contraception in the United States: a qualitative study | Yes | Stated women. | No | Yes | No | Yes | Yes Urban. | No | Yes | No | No | When participant would like to have a baby, and pregnancy history. |
| From request to dispensation: how adolescent and young adult females experience access to emergency contraception in pharmacies | Yes | Stated females. | No | No | No | Yes | Not clear. | No | No | No | No | Age of first emergency contraception request. |
| How can Primary Care Physicians Best Support Contraceptive Decision Making? A Qualitative Study Exploring the Perspectives of Baltimore Latinas | Yes | Stated females. | No | Yes | No | Yes | Yes Urban. | No | No | No | No | Preferred language, and country if birth. |
| Minors' Experiences Accessing Confidential Contraception in Texas | Yes | Identified as female. | Yes | Yes | No | Yes | Yes. Varying community sizes. | No, but did talk about sexual activity. | No | No | No | Sexual activity, insurance, and transportation. |
| Perspectives on family planning services among adolescents at a Boston community health center | Yes | Female gender. | No | Yes | Yes | Yes | Yes Urban. | No, but did talk about sexual partners. | No | No | No | Pregnancy state, family planning clinic uses, pregnancy termination, and sexual activity. |
| Primary care physicians' concerns may affect adolescents' access to intrauterine contraception | N/A Urban family physicians, pediatricians, and obstetrician-gynecologists. | N/A Urban family physicians, pediatricians, and obstetrician-gynecologists. | N/A Urban family physicians, pediatricians, and obstetrician-gynecologists. | N/A Urban family physicians, pediatricians, and obstetrician-gynecologists. | N/A Urban family physicians, pediatricians, and obstetrician-gynecologists. | Yes | Yes Urban. | N/A Urban family physicians, pediatricians, and obstetrician-gynecologists. | N/A Urban family physicians, pediatricians, and obstetrician-gynecologists. | N/A Urban family physicians, pediatricians, and obstetrician-gynecologists. | N/A Urban family physicians, pediatricians, and obstetrician-gynecologists. | Care provider characteristics, female, IUD education in residency, and current clinical practice with intrauterine contraception and adolescents. |
| Racial and Ethnic Discrimination, Medical Mistrust, and Satisfaction with Birth Control Services among Young Adult Latinas | Yes | Stated women. | No | Yes | Yes | Yes | Yes Rural. | Yes | Yes | Yes | No | Acculturation, insurance status, barriers to accessing services, medical mistrust, and everyday discrimination. |
| Seeking the female (internal) condom in retail pharmacies: Experiences of adolescent mystery callers | Adolescent mystery callers posing as 16 year olds. | Stated males and females. | N/A -Mystery caller | N/A, -Mystery caller | N/A, -Mystery caller | Yes | Not clear. Stated across states. | N/A, -Mystery caller | N/A, -Mystery caller | N/A, -Mystery caller | N/A, -Mystery caller |  |
| The Perfect Storm: Perceptions of Influencing Adults Regarding Latino Teen Pregnancy in Rural Communities | N/A -Variety of community stakeholders | N/A -Variety of community stakeholders | N/A -Variety of community stakeholders | N/A -Variety of community stakeholders | N/A -Variety of community stakeholders | Yes | Yes Rural. | N/A -Variety of community stakeholders | N/A -Variety of community stakeholders | N/A -Variety of community stakeholders | N/A -Variety of community stakeholders | Stakeholder demographics: job description, age, gender, education level, religion, race and ethnicity, and nationality. |
| Understanding Barriers to Contraception Screening and Referral in Female Adolescents and Young Adults with Cancer | N/A -Oncology clinicians. | N/A -Oncology clinicians. | N/A -Oncology clinicians. | N/A -Oncology clinicians. | N/A -Oncology clinicians. | Yes | Yes Urban. | N/A -Oncology clinicians. | N/A -Oncology clinicians. | N/A -Oncology clinicians. | N/A -Oncology clinicians. | Clinician demographics: age, race, religion, clinician role, clinician speciality, and years of work at the cancer centre. |
| Understanding Commercially Sexually Exploited Youths' Facilitators and Barriers toward Contraceptive Use: I Didn't Really Have a Choice | Yes | Gender | No -but did ask about sexual partners. | Yes | Not clear. | Not clear. | Yes Urban. | No | No | No | No | Housing status |
| Understanding the low uptake of long-acting reversible contraception by young women in Australia: A qualitative study | Yes | Only stated women. | No | No | No | Yes | Yes. Varying community size (metropolitan and regional) | No | Yes | No | No | Immigration, parental status. Healthcare practitioner demographics: type of healthcare practitioner, residency, and female. |
| Unmet demand for short-acting hormonal and long-acting reversible contraception among community college students in Texas | Yes | Only stated female. | Not clear -must have had sexual intercourse with a man. | Yes | Yes | Yes | Not clear. The community size of South and West Texas are not clear. | Yes | Yes | No | No | Insurance type and usual source of care. |
| Women's perceptions of pharmacist-prescribed hormonal contraception | Yes | Only said women. | No | Yes | No | Yes | Not clear. Community sizes within Central Indiana are not clear. | No | No | No | No | Current number of daily medications, health insurance for prescription medications, and health insurance for birth control. |
| Youth Perspectives on Pharmacists' Provision of Birth Control: Findings From a Focus Group Study | Yes | Only said women. | No | Yes | No | Yes | Yes Urban. | No | Yes | No | No | Health insurance status,  nativity status, and birth control use. |
